# Review on carbonaceous materials and metal composites in deformable electrodes for flexible lithium-ion batteries

**DOI:** 10.1039/d0ra10229f

**Published:** 2021-02-03

**Authors:** Jahidul Islam, Faisal I. Chowdhury, Join Uddin, Rifat Amin, Jamal Uddin

**Affiliations:** Department of Chemistry, University of Chittagong Chittagong 4331 Bangladesh faisal@cu.ac.bd jahid.cu.ctg@gmail.com; Department of Physics, University of Chittagong Chittagong 4331 Bangladesh; Center for Nanotechnology, Department of Natural Sciences, Coppin State University Maryland USA

## Abstract

With the rapid propagation of flexible electronic devices, flexible lithium-ion batteries (FLIBs) are emerging as the most promising energy supplier among all of the energy storage devices owing to their high energy and power densities with good cycling stability. As a key component of FLIBs, to date, researchers have tried to develop newly designed high-performance electrochemically and mechanically stable pliable electrodes. To synthesize better quality flexible electrodes, based on high conductivity and mechanical strength of carbonaceous materials and metals, several research studies have been conducted. Despite both materials-based electrodes demonstrating excellent electrochemical and mechanical performances in the laboratory experimental process, they cannot meet the expected demands of stable flexible electrodes with high energy density. After all, various significant issues associated with them need to be overcome, for instance, poor electrochemical performance, the rapid decay of the electrode architecture during deformation, and complicated as well as costly production processes thus limiting their expansive applications. Herein, the recent progression in the exploration of carbonaceous materials and metals based flexible electrode materials are summarized and discussed, with special focus on determining their relative electrochemical performance and structural stability based on recent advancement. Major factors for the future advancement of FLIBs in this field are also discussed.

## Abstract 2

With the rapid propagation of flexible electronic devices, flexible lithium-ion batteries are emerging as the most promising energy supplier among all of the energy storage devices due to high energy and power densities with good cycling stability.

## Graphical abstract

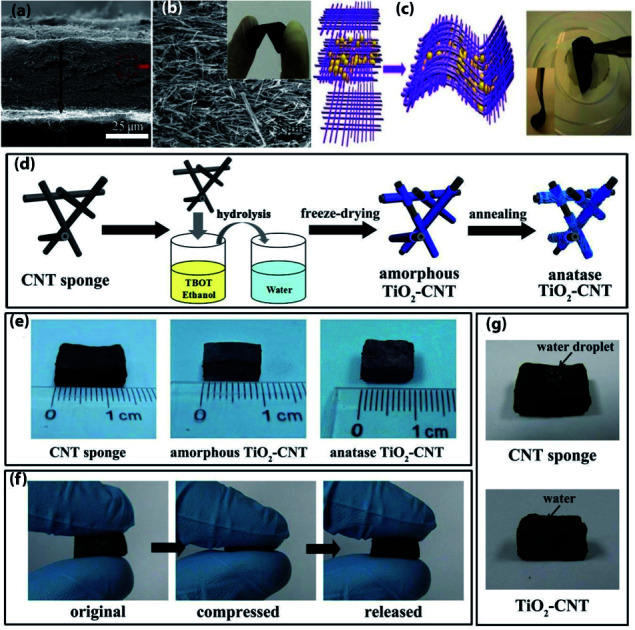

## 1. Introduction

The emergence and instantaneous increase of compact wearable electronic appliances, pliable smartcards, wireless sensors, electronic skin, flexible smartphones, roll-up displays, portable and implantable medical apparatus, *etc.* are receiving enormous attention in the field of flexible energy storage devices.^1–5^ The most typical challenge that is impeding the pragmatic implementation of flexible devices is the attainability of reliable and flexible energy sources with a high rate performance, desirable energy density, and long cycling stability. To be applicable, these electronic systems seek a flexible, small, and lightweight energy storage device that is robust under folding, twisting, bending, stretching, and compressing flexions without compromising their performance and functions. Among all the flexible energy storage systems, FLIBs are highly promising due to their greater power and energy density, longer cycle life, and environmental compatibility.^5–7^

Generally, almost all types of electrodes require conductive materials for the continuous electron flow through the electrodes and maintaining the connection with the outer circuit. Over the past decade, carbonaceous materials (CMs) and metals have attracted researchers' attention due to their abundant sources, high conductivity, structural stability, and suitable mechanical strength.^8–10^ Also, CMs have a large specific surface area, a low rate of resistance rising, and greater energy density with good structural stability after mechanical flexion, and metals excellent electrical conductivity.

Typically, flexible electrodes still stay backward compared with their rigid analogues in terms of electrochemical performance. Moreover, under outer pressure and after long term electrochemical cycling, battery components especially electrode materials undergo permanent morphological distortions such as crack formation, delamination of active materials, or corrosion of conductive current collectors, which can severely affect the innate structure of the electrode materials along with electrolyte/electrode transportation.^11–13^ Moreover, the challenges associated with CMs based flexible electrodes are low specific capacity, costly, not-enough energy density, aggregation of carbon nonmaterial.^14^ Metals undergo fast crack formation, corrosion, poor adhesion with active materials, overweight, low energy, and power densities.^15,16^ Additionally, challenges along with both materials based pliable current collectors are achieving excellent mechanical characteristics such as roughened surface, low density, and tunable thickness.^17^ Therefore, an attempt to morphological modifying of conductive materials and active particles can help to overcome some of these impediments to attaining some degrees of improved flexibility and electrochemical performance.^18^

In this review, we provide a general overview of CMs and metals based flexible electrodes for FLIBs. Also, based on recent works, we tried to find out their relative electrochemical and mechanical performances. Moreover, perspectives for upcoming advancement in this field are also provided.

## 2. Carbonaceous materials supported flexible electrodes

### 2.1. Carbon nanotube (CNT) supported electrodes

**Fig. 1:**
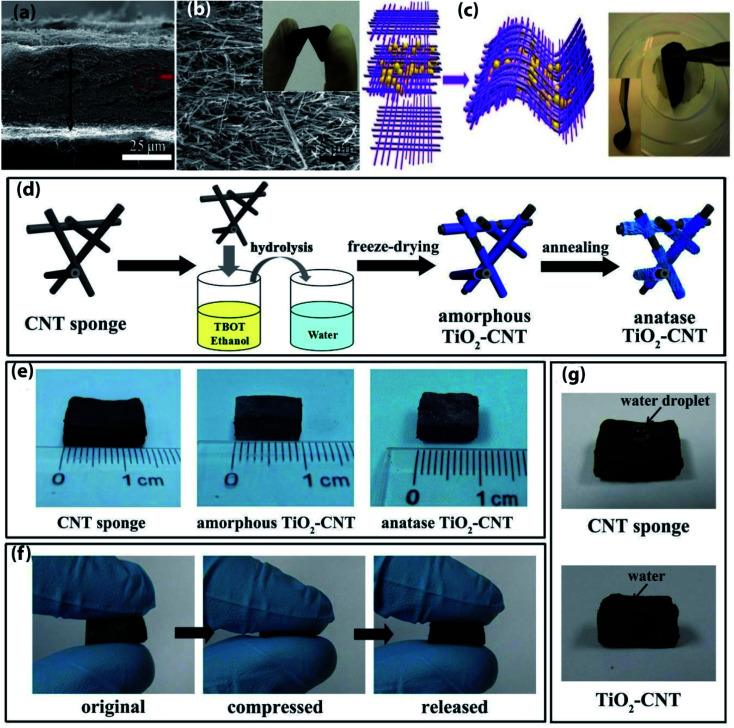
(a) Side-view SEM image of the V_2_O_3_@C NWs film, (b) high-resolution side-view SEM image of the V_2_O_3_@C NWs film and corresponding digital picture. (a and b) Reprinted from ref. 24. (c) Schematic illustration of the fabrication process of the LiNi_0.5_Mn_1.5_O_4_/MWCNT electrodes and digital photo of the LiNi_0.5_Mn_1.5_O_4_/MWCNT network film to show the flexibility. Reprinted from ref. 45. (d) schematic of synthetic process of amorphous and anatase TiO_2_-CNT sponges, (e) photos of an original bulk CNT sponge, an amorphous and an anatase TiO_2_-CNT sponge, (f) compression and recovery process of an anatase TiO_2_-CNT sponge, (g) water droplet staying on the surface of a CNT sponge and soaked into a TiO_2_-CNT sponge. (d–g) Reprinted from ref. 42.

Recently, CNT and CNT-based composites draw great attention to use as electrode materials for flexible LIBs. Owing to excellent mechanical stiffness (Young's modulus of SWCNT and MWCNT are 1 TPa and 0.9 TPa, respectively),^19,20^ remarkable mechanical resiliency, good current carrying capacity and electrical conductivity,^21^ high theoretical capacity, good chemical stability, and improved electrochemical performance than metal current collectors.^16^ Thus, CNT has become the most attractive material for constructing flexible electrodes for LIBs. A variety of procedures have been designed to fabricate CNT-based flexible composite electrodes for LIBs (Fig. 1 and Table 1), including chemical vapor deposition (CVD),^22^ vacuum filtration,^23^ hydrothermal method,^24^ solvothermal,^25^ electrodeposition,^26^ and drop-casting.^27^ For example, by modifying a generally used CVD technique, CNTs are grown into the graphitic carbon layer (CL) that can be served as a flexible and free-standing anode material for LIBs.^28^ The 3D conductive network of CNT synergistically combined with the conductive carbon layer and provides stable electrical conductivity. The film showed no observable degradation even after the film rolled around a glass tube. Thus, the anode maintained a reversible capacity of 572 mA h g^−1^ after 100 cycles at a current density of 0.2 mA cm^−2^. Core-sheath structured nitrogen-doped CNT (N-CNT) film anode materials are synthesized *via* CVD method using acetonitrile as a precursor of nitrogen and carbon under a high temperature of 1060 °C, which has been demonstrated improved mechanical performance and capacity than bare CNT film with a superior electrical conductivity of 410 S cm^−1^.^29^ As a result, N-CNT films delivered a high capacity of 390 mA h g^−1^ at a high rate of 4C and retained 320 mA h g^−1^ after 400 cycles. Also, the electrical resistance of this film is almost the same after 500 times of bending. Light-weight and self-standing pyridine modified boron-doped CNT (Py-B-CNT) film anode materials for flexible LIBs are synthesized through the floating catalyst chemical vapor deposition (FCCVD) method.^30^ The film can tolerate up to 50% strain without any fracture and can recover its initial state. Moreover, the anode exhibited a high initial discharge capacity (1182 mA h g^−1^ at 100 mA g^−1^), good rate performance (167 mA h g^−1^ at 2 A g^−1^), and excellent cyclic stability (548 mA h g^−1^ at 100 mA g^−1^ after 300 cycles). Free-standing Si nanoparticles/MWCNT (weight ratio 3 : 2) composite papers (high mass loading of 3 mg cm^−2^) are prepared by ultrasonication followed by filtration under positive pressure that can be employed as a flexible anode for high energy LIBs.^31^ The 3D network of MWCNT ensures high electrical conductivity and suppresses the volume change of Si particles. So, the composite anode maintained a high discharge capacity (2299 mA h g^−1^ at 100 mA g^−1^), better rate capability (912 mA h g^−1^ at 1000 mA g^−1^), and good cycling performance (1300 mA h g^−1^ at 100 mA g^−1^ after 100 cycles). Si particles incorporated with CNTs (mass loading of about 1.5 mg cm^−2^) are also produced as ultrathin, paper-like, and free-standing anodes for flexible LIBs with high volumetric capacity *via* conformal electrodeposition of Si thin layer on CNTs films for 6 hours.^32^ The porous structure of CNTs film accommodating volume expansion of Si improving electrolyte infiltration simultaneously. The composite film shows the highest volumetric capacity of ∼1400 mA h cm^−3^ at a current density of 200 mA g^−1^ and that can be retained 567 mA h cm^−3^ at 800 mA g^−1^ after 50 cycles. One-step floating catalyst chemical vapor deposition (FCCVD) process in a vertical tube furnace at 1150 °C has been carried out to found free-standing and flexible SiO_*x*_/few walled CNT composite film anodes for high capacity flexible LIBs.^33^ The synergistic effect between high capacity SiO_*x*_ and conductive CNT enables excellent flexibility and electrochemical performances such as exhibit a high reversible capacity (1600 mA h g^−1^ at 30 mA g^−1^) as well as outstanding rate performance with cycling retention (400 mA h g^−1^ at 3 A g^−1^ after 500 cycles). This composite film can withstand extreme structural distortion such as bent, fold, rolled, and even complex origami form without suffering from any configurational failure, makes it an effective electrode in next-generation flexible LIBs.

**Table 1:** CNT based flexible electrodes

Electrode materials	Preparation method	Test process	Performance	Ref.
Peapod-like V_2_O_3_ NRs/CNTs	Hydrothermal	Li metal/coin-cell/0–3 V	186 mA h g^−1^/100 mA g^−1^/125	24
SnS_2_ tubular nanosheath/CNT sponge	Solvothermal	Li metal/coin-cell/0.01–3 V	502 mA h g^−1^/100 mA g^−1^/100	25
Co_3_O_4_/SACNT	Pyrolysis method	N/A	910 mA h g^−1^/0.1C/50	34
MnO_2_ nanowires/MWCNTs	Hydrothermal	Li metal/coin-cell/0.1–3 V	88% capacity retention after 60 cycles	35
Nanosized Fe_2_O_3_/SWCNT	Floating catalyst CVD + oxidation	Li metal/coin-cell/0.001–3 V	801 mA h g^−1^/50 mA g^−1^/90	36
Sodium 1,4-dioxonaphthalen-2-sulfonate/MWCNTs (NQS/MWCNTs)	Dissolution–recrystallization	Li metal/coin-cell/1.6–3.5 V	96% of capacity retention after 50 cycles	37

Thin and flexible 3D hierarchical core–shell SnO_2_@a-Si/CNT hybrid paper (mass loading of 5.9 mg cm^−2^) anodes are fabricated *via* two-step process; first, SnO_2_ nanowires (NWs) were grown on CNT paper by Au catalyzed thermal evaporation, subsequently thin layer of Si was deposited on SnO_2_ NWs by using radio frequency plasma enhanced chemical vapor deposition (RF-PECVD) technique.^38^ In this unique core–shell nanostructure, CNT provides high conductivity and mechanical flexibility, Si served as a high capacity active material, and SnO_2_ NWs prevent Si layer from pulverization, which leads to high initial discharge capacity (3020 mA h g^−1^ at 250 mA g^−1^), large areal capacity (5.2 mA h cm^−2^ after 25 cycles), and outstanding volumetric capacity (1750 mA cm^−3^ after 25 cycles). 2D layer-based 1T-MoSe_2_/SWCNTs hybridized films are prepared as high-performance anodes for flexible LIBs by solvothermal method.^39^ The composite film possesses excellent mechanical characteristics such as, the maximum extension of 22.3% and breaking strength of 34 MPa of the electrodes. Besides, Mo–O–C bond between MoSe_2_ and SWCNT enables great structural stability and stimulates high ions/electrons transportation, which leads to high discharge capacity (1495 mA h g^−1^ at 60 mA g^−1^), excellent rate performance (630 mA h g^−1^ at 3000 mA g^−1^) as well as good cycling durability (971 mA h g^−1^ at 300 mA g^−1^ after 100 cycles). A combination method based on ethylene glycol mediated solvothermal method and vacuum filtration has been proposed to prepared Sb_2_S_3_ nanorods (NRs)/MWCNT free-standing and flexible anodes for LIBs.^40^ The composite anode delivered a high discharge capacity of 930 mA h g^−1^ at a current density of 200 mA g^−1^ and after 100 cycles, at a current rate of 200 mA g^−1^ a capacity was stabilized at 443 mA h g^−1^. NiS nanoparticles electrodeposited on CNT film was utilized in order to obtained NiS NPs/CNT paper-like high capacity flexible LIBs anode.^26^ The composite anode can tolerate the mechanical stress without any structural failure when the film is bent at nearly a 180° angle. This paper-like composite anode delivered a high specific capacity of ∼845 mA h g^−1^ at 60 mA g^−1^ and retained ∼644 mA h g^−1^ after 100 cycles at 300 mA g^−1^ rate. 3D ‘honeycomb-like’ CNT/transition metal oxides (TMOs) (*i.e.* MnO_2_, NiO, Fe_2_O_3_, and Co_3_O_4_) porous microstructured sponges as composite anodes were fabricated *via* freeze-drying and subsequent thermal calcination at 250 °C for 5 h followed by mechanical pressing to decrease the thickness of sponges.^41^ Ultrahigh electrochemical performances can be attributed to the high porosity of electrodes, the large active surface area of CNT sponges, and more open spaces for volume expansion of active particles. Furthermore, a continuous CNT network in 3D architecture can effectively enhance the electron-transfer kinetics, which leads high reversible capacities with good rate performance, for instance, 1104, 1536, 1287 mA h g^−1^ of reversible capacities were delivered by CNT/NiO, CNT/Co_3_O_4_, and CNT/MnO_2_, respectively at the rate of 0.2 A g^−1^. On the other hand, CNT/TMO sponges can recover their initial state after releasing from 70% compressive strain, demonstrating their future demand in special deformable LIBs. A compressible anode, coaxial TiO_2_-CNT sponges were prepared by depositing TiO_2_ onto CVD grown CNT sponges *via* a simple *in situ* hydrolysis method, then freeze-drying followed by annealing at 500 °C.^42^ The highly porous nature of this electrode helps to sufficient amount of electrolyte infiltration and buffer the volume change of TiO_2_, which leads to a high specific capacity of 234 mA h g^−1^ at 50 mA g^−1^, excellent rate performance (149 mA h g^−1^ at 2000 mA h g^−1^), and remarkable cycling stability (210 mA h g^−1^ at 100 mA g^−1^ at the 100^th^ cycles). Moreover, at 91% compressible strain, the anode can be able to sustain structural intactness after releasing from 10 times of volumetric compressed state. The *in situ* synthetic route using rhodanine acetic acid-pyrene (RAAP)-directed growth method has been adopted to prepare a self-weaving CNT-LiNbO_3_ nanoplates system, then decorated with polypyrrole (PPy) layer.^43^ Interwoven highly conductive and porous structure of SWCNT with additional conductive ppy coating synergistically enables high electrochemical kinetics and also helps to maintain structural integrity while retaining almost stable conductivity. Consequently, the electrode can maintain structural integrity with stable conductivity even after 800 repeated bending cycles, and exhibits a high rate performance (175 mA h g^−1^ at 2 A g^−1^) as well as outstanding cycling durability (∼220 mA h g^−1^ at 0.4 A g^−1^ after 500 cycles). V_2_O_5_ nanosheets encapsulated in hollow CNT *via* using electrospinning with CVD process formed nanocables-like structure which is further interwoven in a flexible free-standing and mechanically robust cathode films for high-performance LIBs.^44^ The cathode delivered a high reversible capacity of 224 mA h g^−1^ at 0.15 A g^−1^, surprising rate capability (90 mA h g^−1^ at 30 A g^−1^), and retained 91.7% (211 mA h g^−1^) capacity after 200 cycles at a current density of 0.15 A g^−1^. Light-weight, flexible, and high capacity LiNi_0.5_Mn_1.5_O_4_/MWCNT cathodes for high-power LIBs are synthesized by vacuum filtration,^45^ which can retain 80% capacity at a high current density of up to 2800 mA g^−1^, and until 100 cycles the capacity remaining of 118 mA h g^−1^ at the rate of 1400 mA g^−1^. Impressively, these cathodes provide almost two times higher power density than that of a conventional one. An organic sulfur-linked carbonyl-based poly(2,5-dihydroxyl-1,4-benzoquinoyl sulfide) (PDHBQS)/SWCNT have been proposed as high rate cathodes for flexible LIBs are prepared by vacuum filtration.^46^ This composite cathode exhibits an excellent rate performance of 75 mA h g^−1^ at 5000 mA g^−1^ with retaining 89% of its initial capacity at a current density of 250 mA g^−1^ after 500 cycles. Moreover, a large area (28 cm^2^) flexible LIB based on this cathode and lithium foils anodes are assembled, which maintained 88% of its initial capacity even after 2000 repeated bending cycles while continuously illuminating 14 LEDs simultaneously. An acid-assisted vacuum filtration method has been adopted to prepare perylene-3,4,9,10-tetracarboxylic diimide (PDI)/SWCNT cathodes for highly stable organic LIBs using H_2_SO_4_ as a solvent.^47^ The composite cathode only decays 0.014% capacity per cycle at 500 mA g^−1^ over 2000 cycles and also reserved 93 mA h g^−1^ at a high current density of 1000 mA g^−1^. Sandwich-like Li_2_ZnTi_3_O_8_@MWCNT high rate anodes for flexible LIBs are prepared by vacuum filtration with top and down layer consist of MWCNT.^48^ MWCNT formed a 3D porous and conductive network, which enables stereoscopic electron transportation and facilitate more electrolyte infiltration. As a result, the composite anodes delivered a high reversible capacity at a very current rate of 95.2 mA h g^−1^ at 7000 mA g^−1^ after 1000 cycles, demonstrating its great promise to developed flexible and high energy LIBs.

### 2.2. Carbon nanofibers (CNFs)-supported electrodes

**Fig. 2:**
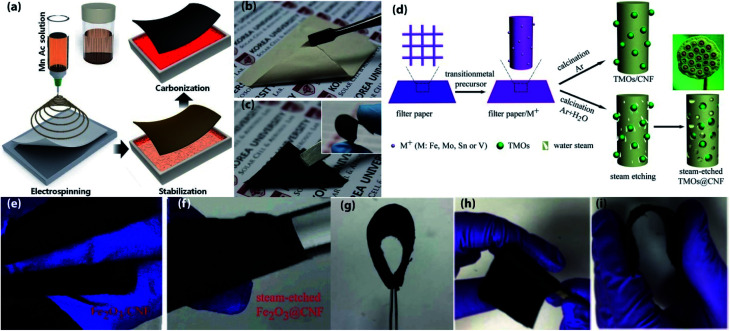
(a) Electrospinning process of preparing CNFs mat, (b) photographs of as-deposited pale orange-colored mat, (c) carbonized freestanding mat with good flexibility. (a–c) Reprinted from ref. 60. (d) Schematic diagram of the fabrication processes of steam-etched TMOs@CNF and TMOs/CNF, (e) broken photographs of Fe_2_O_3_/CNF after bending, (f) photographs of steam-etched Fe_2_O_3_@CNF wrapped around the glass rod. (d–f) Reprinted from ref. 79. (g-i) Photographs of a flexible Si/SiO_2_/C nanofiber mat. Free-standing, flexible Si/SiO_2_/C nanofibers can be folded without any structural damage. Reprinted from ref. 62.

Owing to the large surface area, sufficient electrical conductivity, excellent mechanical strength, 1D configuration with lightweight properties of CNFs have been employed to flexible electrode fabrication.^49,50^ Typically, polyacrylonitrile (PAN) is used as the precursor of CNFs. Generally, one of the most efficient synthesizing techniques involving three steps: electrospinning, stabilization in air, and subsequently high-temperature carbonization has been a widely applied strategy for producing CNFs.^51^ As pristine CNFs demonstrated poor electrochemical performance, in order to improve electrochemical performance several structural modified CNFs and CNFs based composite electrodes have been explored (Fig. 2 and Table 2). For instance, Self-supported and highly porous CNFs (HPCNFs) as flexible anodes for high-performance LIBs are fabricated *via* electrospinning followed by a post-two-step carbonization process, during carbonization of PAN at 1000 °C under Ar atmosphere, a certain amount of air introduced in Ar flow so that CNFs can partially burnt-off and produced a highly porous structure with numerous micropores and mesopores.^52^ The highly porous and 3D interconnected structure of this anode facilitates fast ion diffusion while improving flexibility. As a result, the flexible anode exhibited high reversible capacity (1780 mA h g^−1^ at 50 mA g^−1^ after 40 cycles), superior rate performance (200 mA h g^−1^ at 25 A g^−1^), and ultra long cycle life (1550 mA h g^−1^ at 500 mA g^−1^ after 600 cycles). Ge NPs encapsulated CNFs have been fabricated by electrospinning then stabilized in the air followed by carbonization at 650 °C under Ar/H_2_ atmosphere, which can be served as self-supported flexible anodes for high-performance LIBs.^53^ 3D porous interconnected structure of this hybrid anode facilitates high electrons and ions transportation and enhances electrolyte access toward active materials, which leads to high reversible capacity (∼1420 mA h g^−1^ at 0.15C after 100 cycles), and superior cycle life (829 mA h g^−1^ after 2500 cycles at 1C). 3D flexible core (Si)–shell (Si_3_N_4_) (Si@Si_3_N_4_)/CNFs composite anodes are prepared by electrospinning subsequently stabilized in the air followed by heat treatment at 1200 °C for 3 h under N_2_ atmosphere.^54^ In this 3D configuration, highly conductive CNFs are cross-linked with each other and created numerous void spaces which facilitate fast Li-ion transfer and buffer the volume changes during cycling, results in high discharge capacity (661 mA h g^−1^ at 10 A g^−1^), outstanding rate performance (145 mA h g^−1^ at 80 A g^−1^) and ultra long cycle life (369 mA h g^−1^ at 10 A g^−1^ after 2000 cycles). Free-standing Fe_2_O_3_–SnO_*x*_/CNFs (Fe : Sn ratio of 3 : 1) composite anodes are prepared by electrospinning, then stabilized in the air followed by annealing at 700 °C for 2 h under Ar atmosphere,^55^ in which CNFs are relaxing the volume expansion and shortening the Li-ion diffusion distance while SnO_*x*_ prevents the aggregation of Fe_2_O_3_. As a result, the composite anode delivered a high reversible capacity of 756 mA h g^−1^ after 55 cycles at 100 mA g^−1^. Flexible TiO_2_/SiO_2_/CNFs composite anodes are synthesized through one-spinneret electrospinning followed by calcination at 900 °C.^56^ In this architecture, SiO_2_ improves capacity and TiO_2_ helps to maintain structural integrity and carbon ensures high conductivity and mechanical flexibility. Thus, the composite anode delivers a high rate capability (115.5 mA h g^−1^ at 8 A g^−1^), and superior cycling stability (380.1 mA h g^−1^ at 200 mA h g^−1^ after 700 cycles). *In situ* assembly of 3D MoS_2_ nanoleaves/bacterial cellulose (BC) derived CNF composites are produced through hydrothermal reaction followed by carbonization at 350 °C for 1 h then increased to 800 °C for 1 h.^57^ In this 3D hierarchical architecture, CNFs formed interweaved porous structure which provides highly conductive channels for electrons and Li^+^ diffusion, and MoS_2_ nanoleaves offer more active sites for Li^+^ storage. Consequently, this composite anode exhibits a high discharge capacity (1313 mA h g^−1^ at 0.1 A g^−1^), and ultrahigh cycling durability (581 mA h g^−1^ after 1000 cycles even at a high rate of 1 A g^−1^). On account of, improve the capacity of MoS_2_ based electrode, a hierarchical self-supported core–shell TiO_2_–MoS_2_/CNF mats as flexible anodes has been produced by using a combination of electrospinning, carbonization, and hydrothermal method.^58^ So, the composite anode shows enhanced specific reversible capacity (∼1460 mA h g^−1^ at 100 mA g^−1^), better rate performance (928 mA h g^−1^ at 2 A g^−1^), and long-life cycle duration (1072 mA h g^−1^ over 1000 cycles at 1 A g^−1^), makes it a potential anode materials for the application in advanced flexible LIBs. A novel SnSe/CNFs membrane as a flexible anode has been successfully prepared by electrospinning and subsequent thermal annealing, which can tolerate bending stress when the bending angle is 180°.^71^ The 3D porous and conductive structure is consisted of CNFs, which facilitates high electronic and ionic transportation, and lead to excellent rate performance (384 mA h g^−1^ at 4000 mA g^−1^), and superior cycle life (405 mA h g^−1^ at 1000 mA g^−1^ after 500 cycles). Fe_3_O_4_/CNFs as flexible anodes for LIBs are synthesized *via* using electrospinning, then stabilized in the air followed by heat treatment at 600 °C in N_2_ atmosphere,^72^ in which CNFs successfully buffer the volume expansion of active particles while providing a conducive and flexible scaffold for this composite electrode, results in better discharge capacity (1161 mA h g^−1^ at 0.5 A g^−1^), excellent cycling retention (762 mA h g^−1^ at 0.5 A g^−1^ and 611 mA h g^−1^ even at 1 A g^−1^, over 300 cycles). Flexible ultra-long, aligned CNFs embedded with In_2_O_3_ nanocrystals has been prepared by electrospinning, subsequent air stabilization followed by calcined at 550 °C for 5 h under N_2_ atmosphere, which can be used as a flexible anode material for thin and high-performance LIBs.^73^ Ultrafine In_2_O_3_ particles can shorten the diffusion distance of Li-ion, and encapsulation of In_2_O_3_ in CNFs matrix helps to direct exposure of active particles in the electrolyte and formed a stable and uniform SEI layer and also reduce pulverization lead to a high electrochemical performance and cycling stability. As a result, the composite anode demonstrated a high discharge capacity of 282.2 mA h g^−1^ at 1000 mA g^−1^ after 1000 cycles, and a capacity of 183.7 mA h g^−1^ at a high current rate of 10 000 mA g^−1^ even after 2900 cycles. Since, the improvement in conductivity, flexibility, and Li^+^ storage sites of CNFs, nitrogen doping has been proposed as an effective strategy. For instance, polyimide (PI) derived N-CNFs membranes as flexible anode materials for high-performance LIBs are prepared by electrospinning, then imidization, and subsequent carbonization at various temperatures 550–950 °C for 0.5–10 h.^74^ The film which carbonized at 650 °C for 3 h demonstrated the highest reversible specific capacity (695 mA h g^−1^ at 0.1 A g^−1^), and better cycling stability (245 mA h g^−1^ after 300 cycles even at 1.5 A g^−1^). Robust N-doped CNF film uniformly encapsulated amorphous SiO_2_ NPs as free-standing and flexible anodes for LIBs are prepared by electrospinning, air stabilization followed by carbonization at 800 °C for 2 h under Ar/H_2_ (95 : 5; v/v) atmosphere.^75^ Due to the encapsulation of active particles, CNFs can prevent the aggregation and pulverization and also suppress the volume expansion of active particles. So, the anode exhibits a high reversible capacity of 405 mA h g^−1^ at 500 mA g^−1^ after 1000 cycles and maintained structural integrity during the bending with a highest bending angle of 180°. In order to improve self-volume buffering properties and mechanical stability under long term flexion, porous SiO_2_ nanoclusters have been suggested as the active material. So, porous-SiO_2_ within N-CNFs as flexible anodes are prepared by electrospinning, and subsequently stabilized in the air followed by carbonization at 800 °C under N_2_ atmosphere for 2 h,^76^ in which porous-SiO_2_ buffer the volume expansion by itself and the deformable anode can maintain its favorable electrochemical performance after 1000 times of bending with the largest bending angle of 180°. Furthermore, after 1000 times of charge–discharge cycles, the charge transfer resistance surprisingly decreased significantly, indicates the long-term stability of SEI layer and fast diffusion kinetics of Li ions. Therefore, this could be an effective route to develop SiO_2_ based flexible and highly stable anode materials for LIBs. Flexible Fe_3_O_4_ NPs/N-doped CNFs as hybrid anodes for LIBs are synthesized *via* electrospinning, then air stabilization followed by carbonization at 600 °C for 2 h in N_2_ atmosphere.^77^ The synergistic effect between Fe_3_O_4_ and CNFs with a 3D interlinked conductive network of CNFs can ascribe the high electrochemical performance of this hybrid anode. Thus, the anode delivered a capacity of 522 mA h g^−1^ at 0.1 A g^−1^ after 200 cycles, and superior rate performance (407 mA h g^−1^ at 5 A g^−1^). Hierarchical CuO_*x*_–Co_3_O_4_ heterostructure nanowires decorated on porous N-CNFs as free-standing anodes for high-performance flexible LIBs are fabricated by using electrospinning, solvothermal method, and heat treatment at 400 °C for 2 h in N_2_ atmosphere then annealing at 180 °C for 6 h in air.^78^ The impressive electrochemical performance can be attributed to the rational modeling of electrode configuration and the noticeable contributions from CuO_*x*_–Co_3_O_4_ heterostructure nanowires as well as the 3D conductive CNFs scaffold, which can enhance the interfacial contact, shorten the Li^+^ diffusion distance, and improve the active sites for Li storage. As a result, the hybrid anode exhibits a high discharge capacity of 1122 mA h g^−1^ at 200 mA g^−1^ after 100 cycles and, an excellent reversible capacity of 668 mA h g^−1^ after 1000 cycles at 2 A g^−1^. An effective steam etching strategy has been developed to improve flexibility and capacity of CNFs based electrodes, such as a series of TMOs (V_2_O_3_, Fe_2_O_3_, MoO_2_, and SnO_2_) are used to record their performances, all of them are fabricated by the same process.^79^ First, filter papers are soaked with TMO precursors then drying, finally, calcined for 3 h, during calcination water streams are introduced for 0.5 h after 1 h of calcination for the sake of creates numerous holes in filter paper derived CNFs skeleton. In this system, steam-etched TMOs@CNFs demonstrated lotus seed-pod like structure which is beneficial for mitigating volume change and pulverization, and numerous holes in CNFs skeleton facilitates high Li^+^ access towards active particles also absorbed the stress-induced from mechanical deformation results the films are successfully wrapped around a glass rod without any structural failure. Consequently, all of them retained over 100% of their initial capacity after 1500 cycles even at a high rate of 2 A g^−1^, indicates the high effectiveness of this unique strategy.

**Table 2:** CNFs based flexible electrodes

Electrode materials	Preparation method	Test process	Performance	Ref.
MnO NPs/CNFs	Electrospinning + thermal carbonization	Li metal/coin-cell/0.01–3 V	655 mA h g^−1^/0.5 A g^−1^/280	59
MnO nanocrystals/CNFs	Electrospinning + carbonization	Li metal/coin-cell/0.01–3 V	923 mA h g^−1^/123 mA g^−1^/90	60
Carbon coated Ge NWs/CNFs	Vapor–liquid–solid (VLS)/electrospinning + carbonization	Li metal/coin-cell/0.005–3 V	∼840 mA h g^−1^/2C/200	61
CVD carbon-coated Si/SiO_2_/CNFs	Electrospinning + carbonization + CVD	Li metal/coin-cell/0.01–2 V	733 mA h g^−1^/100 mA g^−1^/50	62
Sn NPs/CNFs	Electrospinning + carbonization	Li metal/coin-cell/0.01–3 V	460 mA h g^−1^/200 mA g^−1^/200	63
SnO_*x*_–ZnO/CNFs	Electrospinning + annealing	Li metal/coin-cell/0–3 V	963 mA h g^−1^/100 mA g^−1^/55	64
Porous carbon nanofiber (PCNF)@MoS_2_ NSs	Electrospinning + solvothermal reaction	Li metal/coin-cell/0.01–3 V	736 mA h g^−1^/0.05 A g^−1^/50	65
CNFs@NiS NPs	Electrospinning + carbonization + chemical bath deposition + sulfidation	Li metal/coin-cell/0.01–3 V	805.8 mA h g^−1^/0.1 A g^−1^/100	66
Fe_3_O_4_ NPs/CNFs aerogels	Freeze-drying + hydrothermal treatment + carbonization	Li metal/coin-cell/0.005–3 V	755 mA h g^−1^/0.1 A g^−1^/80	67
WO_*x*_-CNFs	Electrospinning + heat treatment	Li metal/coin-cell/0.01–3 V	321 mA h g^−1^/500 mA g^−1^/85	68
NiCo_2_O_4_ nanosheets/carbon fibers	Two-step heat treatment + hydrothermal treatment	Li metal/coin-cell/0.01–3 V	1128 mA h g^−1^/100 mA g^−1^/80	69
SnO_2_/N-doped CNFs	Electrospinning + carbonization	Li metal/coin-cell/0.01–3 V	754 mA h g^−1^/1 A g^−1^/300	70

### 2.3. Graphene-supported based electrodes

**Fig. 3:**
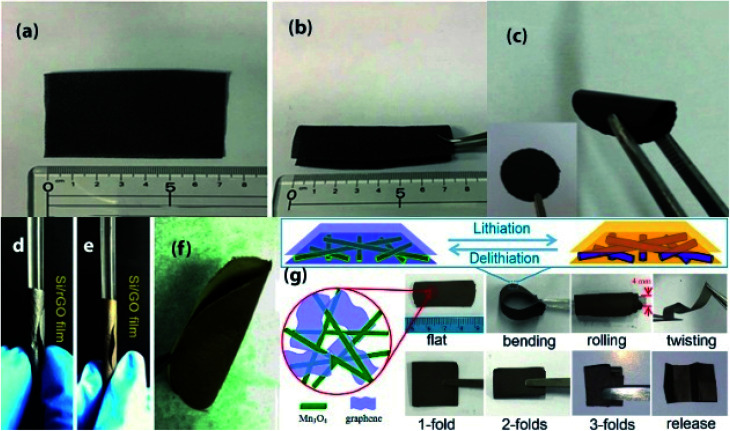
(a and b) Photographs of a flexible Ge-QD@NG/NGF yolk–shell electrode. Reprinted from ref. 87. (c) Photograph of the flexible Bi_2_Se_3_/graphene film. Reprinted from ref. 97. (d–f) Exhibition of Si/rGO flexible films in different states. Reprinted from ref. 90. (g) Schematic diagram (top view and cross-sectional view) of the rGO/Mn_3_O_4_ membrane and its digital photos illustrating the flexibility (bending, rolling, twisting) and foldability (1-, 2-, 3-fold) states. Reprinted from ref. 96.

Graphene is a 2D sp^2^-bonded single layer of carbon sheet has excellent charge carrier mobilities (about 10 000 cm^2^ V^−1^ s^−1^) at a room temperature, high electrical conductivity (4.8 × 10^2^ S cm^−1^), large surface area (2630 m^2^ g^−1^), intrinsic mechanical flexibility, has zero effective mass, good chemical robustness, and light-weight in properties.^80–83^ Based on those useful properties many efforts have been devoted to the fabrication of graphene-based flexible electrodes (Fig. 3 and Table 3). However, graphene-based electrodes possess the low theoretical capacity and suffer high initial irreversible capacity as well as serious capacity fading. So, it is necessary to incorporating high capacitance active materials on the surface of graphene or intercalation between graphene sheets. This section will highlight the recent developments of graphene-based electrodes. For example, a flexible holey reduced graphene oxide (rGO) paper has been suggested for improving rate performance and prepared by a multiple-step process; first, pores forming into the basal plane of GO *via* a wet chemical method combined with ultrasonic vibration and mild acid oxidation, then simple filtration, and subsequent air drying followed by calcination at 700 °C in Ar or H_2_(10%)/Ar(90%) flow.^84^ The film with a thermal reduction in Ar flow shows a larger pore size than that of Ar/H_2_ flow. Moreover, numerous pores in rGO enable high ion diffusion kinetics, abundant Li storage site, and much more accessible interior space which lead to almost no capacity fading after 1000 cycles at a very high current of 5 A g^−1^ and 10 A g^−1^. An interfacially modified Si NPs/graphene foam (GF) monolith as high performance flexible composite anodes are produced by ultrasonication, then freeze-drying followed by calcination at 350 °C.^85^ Highly conductive 3D interconnected graphene foam facilitates rapid ion/electrons transportation, and the confining of Si NPs in GF reduces direct exposure of active particles in the electrolyte which leads to a stable SEI layer. As a consequence, the composite anode exhibited surprisingly high reversible capacity and cycling stability (1198 mA h g^−1^ at 1 A g^−1^ and retained 1170 mA h g^−1^ even after 1200 cycles), and superior rate performance (609 mA h g^−1^ at 8 A g^−1^). Self-standing flexible silicon oxycarbide (SiOC)/graphene composite paper anodes are fabricated by polymer pyrolysis process at 1000 °C followed by vacuum filtration,^86^ which demonstrated high rate performance (∼175 mA h g^−1^ at 2400 mA g^−1^), and ultra-long cycle durability (588 mA h g^−1^ at 1600 mA g^−1^ after 1020 cycles). A 3D interconnected porous nitrogen-doped graphene foam with encapsulated Ge quantum dot/nitrogen-doped yolk–shell nanoarchitecture (Ge-QD@NG/NGF), synthesized by the step-by-step process; which can be used as a high-performance anode for flexible LIBs.^87^ First, N-doped graphene foams are produced by the CVD technique using porous Ni foam as a template. Then, Ge precursor placed in N-doped graphene foam by hydrothermal method followed by annealing at 650 °C in Ar/H_2_ atmosphere for 6 h, finally, acid (1 M HCl) etching to remove Ni template. In this architecture, 3D interconnected unique yolk–shell structure provides numerous open channels for fast Li-ion diffusion and accommodates the volume expansion of Ge by internal void space. As a result, this anode exhibits long-term cycle life (1220 mA h g^−1^ at 1600 mA g^−1^ after 1000 cycles), and exceptional rate performance (792 mA h g^−1^ at a high rate of 40C even after 200 cycles). Also, this hybrid anode can retain ∼95.2% capacity after 20 bending cycles. Pliable high-rate hollow metal oxides (Fe_2_O_3_ and CuO) nanoparticles/porous graphene composite anodes are fabricated through simple filtration followed by a controlled oxidation process.^88^ In this system, hollow metal oxide NPs placed in the pores of graphene sheets and formed Cu/Fe–O–C chemical bond between CuO/Fe_2_O_3_ NPs and graphene generates strong interfacial interaction. On the other side, hollow NPs and porous graphene effectively shorten the diffusion path for Li-ion lead to abnormal rate capability with cycle life. Such as, the specific capacities are (741 mA h g^−1^ and 141 mA h g^−1^ at 10 A g^−1^ and 50 A g^−1^, respectively over 1000 cycles) for Fe_2_O_3_@graphene and (980 mA h g^−1^ and 168 mA h g^−1^ at 10 A g^−1^ and 100 A g^−1^, respectively after 1000 cycles) for CuO@graphene, makes them promising anode materials in commercial flexible LIBs.

**Table 3:** Graphene based flexible electrodes

Electrode materials	Synthesis method	Test process	Performance	Ref.
Folded structured graphene paper	Freeze-drying + thermal reduction + mechanical pressing	Li metal/coin-cell/0.01–3.5 V	568 mA h g^−1^/100 mA g^−1^/100	89
Si hallow nanosheets/rGO	Magnesiothermic reduction + layer-by-layer assembly	Li metal/coin-cell/0.005–2 V	650 mA h g^−1^/200 mA g^−1^/150	90
CuO NSs/rGO	Vacuum filtration + hydrothermal reduction	Li metal/coin-cell/0.01–3 V	736.8 mA h g^−1^/67 mA g^−1^/50	91
Porous Fe_2_O_3_ encapsulated grapheme	Confined Ostwald ripening + thermal treatment	Li metal/coin-cell/0.01–3 V	1129 mA h g^−1^/0.2 A g^−1^/130	92
TiO_2_–grapheme	Dipping + hydrothermal method	Li metal/coin-cell/1–3 V	122 mA h g^−1^/2 A g^−1^/100	93
Rice ball-like ZnCo_2_O_4_/rGO	Solvothermal + vacuum filtration	Li metal/coin-cell/0.01–3 V	908.7 mA h g^−1^/500 mA g^−1^/500	94
Graphene/SnO_2_ nanocomposite paper	Simple filtration + thermal reduction	Li metal/coin-cell/0.005–1.5 V	438.5 mA h g^−1^/100 mA g^−1^/50	95
Graphene/Mn_3_O_4_NWs	Hydrothermal + vacuum filtration	Li metal/coin-cell/0.005–3 V	702 mA h g^−1^/100 mA g^−1^/100	96
Bi_2_Se_3_/graphene	Vacuum filtration + thermal reduction	Li metal/coin-cell/0.001–3 V	203 mA h g^−1^/50 mA g^−1^/100	97

Highly conductive and flexible graphene/modified mesoporous anatase TiO_2_ composite anodes are synthesized by two-step vacuum filtration, the down layer consists of a bare graphene layer obtained by the first filtration, then the top layer deposited as a composite layer.^98^ Numerous macropores in this film and mesopores in TiO_2_ facilitate longitudinal diffusion of the electrolyte and graphene layers provide high electrical conductivity for the hybrid anodes. Thus, the hybrid anode exhibits a better cycling performance (155 mA h g^−1^ at 1C after 200 cycles and ∼94 mA h g^−1^ at a high rate of 5C even after 3500 cycles), and excellent rate performance (76 mA h g^−1^ at 20C). Layered structured graphene/porous ZnCo_2_O_4_ nanosheets composite films are prepared by vacuum filtration followed by heating at 600 °C for 2 h, which has been suggested as flexible anodes for high-performance LIBs.^99^ The porous structure of active nanosheets facilitates fast Li-ion transfer rate and reducing diffusion path, and graphene sheets provide high conductivity for electrodes. As a result, after 1000 cycles, a high specific capacity of 791 mA h g^−1^ was achieved at a high rate of 1 A g^−1^, and superior rate performance (322 mA h g^−1^ even at 8 A g^−1^). 3D layered SnO_2_ quantum dots/graphene frameworks as flexible anodes are fabricated *via* using a combination of hydrothermal method, freeze-drying, and calcination at 300 °C for 2 h in Ar atmosphere.^100^ This 3D unique design with plenty of macropores as well as high specific surface area promotes fast ion/electrons transfer rate, and more active sites for Li binding, which leads to a high reversible capacity (1300 mA h g^−1^ at 100 mA g^−1^), high rate capability with ultralong cycle life (200 mA h g^−1^ at a high rate of 10 A g^−1^ even after 5000 cycles), that is far superior than previously mentioned SnO_2_ based electrode. Porous Mn_3_O_4_ nanorods/rGO hybrid paper as flexible anodes have been produced by simple filtration, and subsequent thermal reduction at 400 °C for 2 h.^101^ In this as formed 3D structures, active materials placed between flexible rGO layers, effectively buffer volume change of Mn_3_O_4_ NRs and prevent pulverization. Moreover, the porous structure of composite film facilitates fast Li-ion diffusion and increases the wettability of electrolytes. Consequently, after 100 cycles, the capacity stabilized at 573 mA h^−1^ at the rate of 100 mA g^−1^ as well as exhibits a good rate performance (196 mA h g^−1^ at 2000 mA g^−1^). Ultralong MnO@C/rGO as 3D hierarchical high-performance anode materials for flexible LIBs are fabricated by vacuum-assisted layer-by-layer assembly of polydopamine coated MnO_2_ NWs and GO, then thermal reduction at 700 °C for 2 h.^102^ In this 3D architecture, coaxial MnO@C NWs multidimensionally interconnected with rGO sheets and formed continuous conductive path and open channels for Li^+^/e^−^ transport results high reversible capacity (920 mA h g^−1^ at 0.2 A g^−1^), long term cycling duration (719 mA h g^−1^ after 800 cycles at 2 A g^−1^), and superior rate performance (396 mA h g^−1^ at 10 A g^−1^). A freestanding mesoporous Li_4_Ti_5_O_12_/rGO nanocomposites membrane as high rate flexible anodes for LIBs has been fabricated through using a combination of hydrothermal treatment, then vacuum filtration followed by thermal annealing at 600 °C for 2 h.^103^ The excellent rate performance of this composite anode can be attributed to the improved Li^+^ transfer through the mesoporous pathways, and high electrical conductivity of rGO sheets. So, the anode shows a good rate performance (135.4 mA h g^−1^ at 7000 mA g^−1^) and retained 93.9% capacity at 1750 mA g^−1^ after 500 cycles. Freestanding and flexible Mn_2_P_2_O_7_-carbon@rGO hybrid films are prepared as high-performance anodes for LIBs. First, Mn^2+^ was absorbed by bacteria, then mixing with GO and subsequent vacuum filtration followed by thermal treatment at 700 °C for 2 h to obtained micro-yolk–shell Mn_2_P_2_O_7_-carbon@rGO hybrid papers.^104^ The framework consists of the graphitized bacterial carbon with rGO not only prevents the agglomeration of Mn_2_P_2_O_7_ but also provides sufficient space for buffer the volume changes of Mn_2_P_2_O_7_ during cycling which leads to high reversible capacity (880 mA h g^−1^ at 100 mA g^−1^), and excellent rate performance with long cycle life (400 mA h g^−1^ at 5000 mA g^−1^ over 2000 cycles). Core–shell Fe_7_S_8_@C NPs encapsulated within three-dimensional graphene composites as flexible high-performance anodes for LIBs are synthesized by excessive metal ion spatially confined Ostwald ripening and vitamin C induced assembly, afterward calcination with Se powder at 600 °C for 2 h, using metal–organic framework/3D graphene as precursor.^105^ This anode delivered a high reversible capacity (884.1 mA h g^−1^ at 0.1 A g^−1^ after 120 cycles), and superior cycling durability (815.2 mA h g^−1^ after 250 cycles at 1 A g^−1^). 3D freestanding cathodes composed of graphene/LiFePO_4_ nanostructures are produced *via* solvent evaporation technique,^106^ which retained 165.3 mA h g^−1^ after 50 cycles at 0.1C. Moreover, after 100 times of bending with a bending angle of 45°, the composite cathode shows only slight decay of discharge capacity, demonstrated its high mechanical stability. Freestanding flexible MoO_3_ nanobelts/graphene composite cathodes were fabricated by applying two-step ultra-fast microwave hydrothermal treatment followed by vacuum filtration,^107^ which exhibits a high initial discharge capacity (291 mA h g^−1^ at 100 mA g^−1^), and after 100 cycles, maintained 172 mA h g^−1^ at 100 mA g^−1^ that is superior to that of bare MoO_3_ based electrodes.

### 2.4. Carbon cloth/fabric/textile (CFT)-supported electrodes

**Fig. 4:**
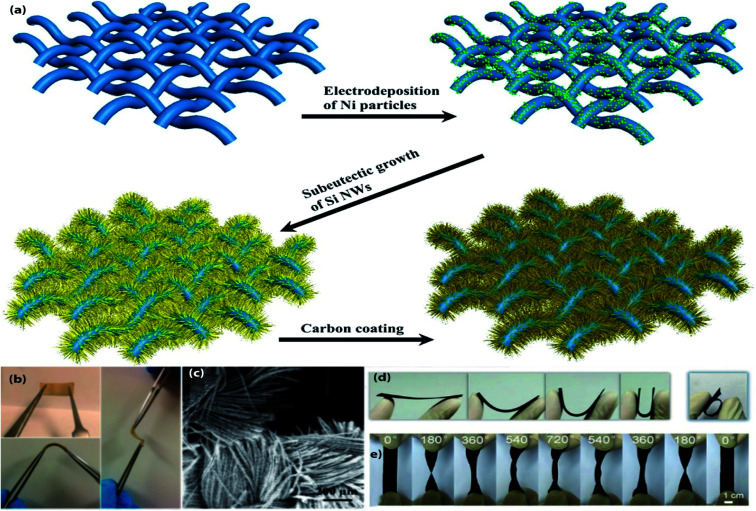
(a) Schematic Illustration of the growth of carbon-coated Si NWs on flexible carbon cloth substrate. (b) Digital image of the c-Si NWs/carbon cloth electrode, (c) representative SEM image of the c-Si NWs/carbon cloth electrode. (a–d) Reprinted from ref. 111. (d and e) Photographs of the flexible CF@SnO_2_-NS@AC electrode during the folding, the rolling, and twisting tests. (d and e) Reproduce with permission,^124^ copyright 2018, Elsevier.

3D CFT has been proposed as promising substrates for constructing flexible electrodes due to high electrical conductivity, good corrosion resistance, excellent mechanical flexibility, lightweight and commercial availability.^108–110^ Depositing, coating, or direct growing high capacity active materials on CFT conductive substrates have been found to be an effective route for fabricating the flexible electrodes. A variety of CFT based flexible electrodes (Fig. 4 and Table 4) have been extensively studied in flexible LIBs. This section will comprehensively present the recent developments in this field. Self-supported carbon-coated Si NWs *in situ* grown on carbon fabric (CF) *via* a nickel-catalyzed one-pot atmospheric pressure CVD process with a mass of 1.2–2.5 mg cm^−2^, which has been proposed as flexible anodes for LIBs.^111^ 1D NWs and CF formed a porous architecture, accommodate the huge volume change as well as increasing electrolyte infiltration. Moreover, an additional carbon layer on Si NWs helps to prevent further oxidation of active NWs by confining active NWs interior of the carbon layer results a high reversible specific capacity (3362 mA h g^−1^ at 100 mA g^−1^ after 100 cycles), better cycling stability (2061 mA h g^−1^ after 500 cycles even at a high current density of 1 A g^−1^), and superior rate performance (1500 mA h g^−1^ at 5 A g^−1^), also the high areal capacity of 5 mA h cm^−2^. 3D ordered macroporous MoS_2_@C/CC as high-performance flexible anodes are fabricated by dipping of CC in active materials precursor solution followed by calcination at 600 °C for 2 h in Ar steam.^112^ The high electrochemical performance of this anode can be attributed to the 3D interconnected ordered microporous nanostructure which not only facilitates large specific active surface area but also promotes the Li^+^ diffusion rate as well as effectively buffers the volume change. As a result, this hybrid anode delivered a high areal capacity (3.428 mA h cm^−2^ at 0.1 mA cm^−2^), rapid charge–discharge performance (1.361 mA h cm^−2^ at 5 mA cm^−2^), and excellent capacity retention (2.471 mA h cm^−2^ after 100 cycles at 0.5 mA cm^−2^). Moreover, after 300 bending cycles, the anode can maintain its structural integrity and initial morphology. To attain a more favorable cycle life with areal capacity, another MoS_2_ contained, hierarchical Fe_2_O_3_@carbon fabric (CF) decorated with MoS_2_ nanosheets as composite anodes for flexible ultrahigh areal capacity LIBs have been synthesized by using a combination of electrospinning, and subsequent calcination followed by hydrothermal treatment.^113^ The enhanced electrochemical performance can be ascribed to the synergistic effect between Fe_2_O_3_@CF and MoS_2_ NSs. Consequently, the anode exhibits outstanding rate capability (304 mA h g^−1^ at 5 A g^−1^), and better cycle life (938 mA h g^−1^ at 0.2 A g^−1^ after 300 cycles). A new 3D hierarchical sandwiched type amorphous carbon-coated-SnO_2_ nanosheets/CC as composite anodes for ultra-flexible LIBs are fabricated by a multiple-step process.^114^ First, SnO_2_ NSs were grown on CC substrate by hydrothermal reaction followed by calcination; afterward, coated with amorphous carbon *via* using another hydrothermal treatment and subsequent annealing, which did not show any observable changes even after 200 continuous bendings. In this system, 2D SnO_2_ NSs with CC constructed a 3D porous architecture with plenty of void spaces, which is beneficial for enabling high active surface area and shortening the diffusion distance of Li^+^ ion. In addition, the extra carbon layer formed a chemical bond with SnO_2_ NSs, reduce detachment and volume expansion induced stress. Consequently, the hybrid anode exhibits a high specific capacity (968.6 mA h g^−1^ at 85 mA g^−1^ after 100 cycles), and long cycle life (471.2 mA h g^−1^ over 1500 cycles at 900 mA g^−1^). Flexible freestanding and high-performance TiO_2_ nanocrystals/CC anodes for LIBs are prepared by oleic acid assisted drop-casting followed by heating at 450 °C,^115^ which can tolerate bending stress when the bending angle was 180° even after 100 times of bending. Also, the small size of active material crystals can not only shorten the diffusion length of Li ions but also provide a high available active surface area for electrochemical reactions. So, the hybrid anode delivered a capacity of ∼150 mA h g^−1^ after 100 cycles at 500 mA g^−1^. A flexible pouch type half-cell was assembled using this anode, which can power a red LED even after 100 times of flexion. A flexible TiO_2_@TiN NWs/CC composite anodes with high rate capability for LIBs are synthesized by a hydrothermal reaction followed by calcination at 800 °C under Ar flow then 5 min in NH_3_ gas, in which the thin TiN layer improve electronic conductivity and electrochemical performance than pristine TiO_2_.^116^ As a result, the composite anode shows a high discharge capacity (579 mA h g^−1^ at 335 mA g^−1^), excellent rate performance (136 mA h g^−1^ at 10.05 A g^−1^), better cycling stability (203 mA h g^−1^ at 3350 mA g^−1^ after 650 cycles), which is far beyond than previously mentioned pristine TiO_2_ contained one. Another TiO_2_ contained, TiO_2_@Ge core–shell nanorods arrays/CT hybrid anode for high-density flexible LIBs are fabricated by step-by-step processes; first, TiO_2_ nanorod arrays hydrothermally grown on CT, then coated with Ge layer using radio frequency magnetron sputtering technique followed by thermal treatment at 450 °C.^117^ The 3D TiO_2_@Ge nanostructure with numerous void spaces and loose texture of CT not only enhances the electrolyte penetration through the electrode with rapid ion diffusivity but also relaxing the volume expansion induced stress. In addition, carbon textile facilitates high electrical conductivity for electrochemical reactions. Consequently, the hybrid anode delivered a high discharge capacity (1603.7 mA h g^−1^ at 1 A g^−1^), outstanding cycle life with better rate performance (700.3 mA h g^−1^ at 5 A g^−1^ over 600 cycles), demonstrated favorable performance than that of the abovementioned TiO_2_ contained electrodes. ZnCo_2_O_4_-urchins/CF as a flexible anode for LIBs are prepared by a hydrothermal approach followed by heat treatment at 400 °C.^118^ The anode demonstrated a high discharge capacity (1310 mA h g^−1^ at 180 mA g^−1^ and 1350 mA h g^−1^ even at a high current density of 4.5 A g^−1^), excellent cycle life (1180 mA h g^−1^ at 180 mA g^−1^ and 750 mA h g^−1^ at a high rate of 18 A g^−1^ over 100 cycles). Self-supported flexible high-performance Zn_2_GeO_4_ nanorods/CC anodes are fabricated *via* simple hydrothermal treatment combined with thermal treatment at 450 °C for 2 h in N_2_ atmosphere,^119^ in which synergistic effect between active nanorods and CC ascribed to the excellent electrochemical performance of these hybrid anodes. So, the hybrid anode delivered a high discharge capacity (1851.9 mA h g^−1^ at 200 mA g^−1^), better rate capability (847.5 mA h g^−1^ at 2000 mA g^−1^), and the reversible capacity stabilized at 1302.3 mA h g^−1^ after 200 cycles at 200 mA g^−1^ rate. A flexible anode composed of mesoporous NiCo_2_O_4_ nanowire arrays uniformly grown on carbon textile by a simple surfactant-assisted hydrothermal approach followed by short post-annealing.^120^ In this architecture, loose texture and mesoporous NiCo_2_O_4_ NWAs provides short electrolyte and ion diffusion channels as well as accommodate the volume change during cycling results high discharge capacity (1524 mA h g^−1^ at 0.2 A g^−1^), good rate capability (778 mA h g^−1^ at 2 A g^−1^), and better cycle life (854 mA h g^−1^ after 100 cycles at 0.5 A g^−1^). A pre-lithiation approach has been applied to resolve the initial capacity fading of NiCo_2_O_4_ based electrodes, thus lithiated NiCo_2_O_4_ NWs/CC hybrid anodes for flexible LIBs are fabricated through a hydrothermal approach followed by calcination at 300 °C in the air for 3 h, then applying pre-lithiation.^121^ Therefore, the pre-lithiated anode maintained a discharge capacity of 1026 mA h g^−1^ in the 10^th^ cycle at a current density of 100 mA g^−1^. In addition, a flexible full cell was assembled using this anode with V_2_O_5_/CC cathode, the cell exhibited an energy density of 364.2 W h kg^−1^ at a power density of 240 W h kg^−1^ with no obvious capacity fading even after 200 folding cycles. A novel Fe_2_N NPs/CT hybrid anode with high power density has been fabricated by hydrothermal reaction followed by calcination at 600 °C in NH_3_ gas for 1 h, which has been suggested for high-performance flexible LIBs.^122^ The large surface area of Fe_2_N NPs and lose texture with high conductivity of CT can facilitate high electron/ions transfer and accommodate the volume change of active particles during electrochemical reaction which leads to attaining high reversible capacity with better capacity retention at a high rate (900 mA h g^−1^ at 6000 mA g^−1^ and retained 76% (240 mA h g^−1^) after 300 cycles). Well-aligned binary lithium reactive zinc phosphide (Zn_3_P_2_) nanowire arrays are grown on carbon fabric by a facile CVD method, which has been proposed as an advanced integrated anode for flexible LIBs.^123^ The hierarchical structure with sufficient void spaces between nanowire arrays is beneficial for more electrolyte penetration, shorten the diffusion length of Li^+^, and buffering the volume change during cycling results in high rate capability (400 mA h g^−1^ at 15 A g^−1^), and long cycle life (1000 mA h g^−1^ after 200 cycles at 400 mA g^−1^). *In situ* oxidized CuO nanosheets/CC composite anodes for high-performance flexible LIBs are produced by a facile two-step method combining magnetron sputtering and solution immersion.^124^ In this hybrid system, carbon cloth provides a highly conductive and pliable scaffold with sufficient open void spaces for buffering the volume change. Also, 2D nanosheets shorten the diffusion distance of Li ions. Hence, the composite anode shows a high capacity with better retention (711.2 mA h g^−1^ at 500 mA g^−1^ and maintained 88% after 100 cycles), as well as high rate capability (448.9 mA h g^−1^ at 2000 mA g^−1^). To obtained hierarchical mesoporous NiO nanosheets/CC flexible anode materials for high-performance LIBs, a hydrothermal treatment afterward calcination process has been adopted,^125^ in which mesoporous NiO NSs interconnected with each other and creates extra void spaces to infiltrate more electrolyte, shorten the diffusion path as well as relaxing the volume change. So, the hybrid anode exhibits a high discharge capacity (1156.5 mA h g^−1^ at 100 mA g^−1^), good rate performance (298.4 mA h g^−1^ at 5000 mA g^−1^), and better cycle life (758.1 mA h g^−1^ over 150 cycles at 700 mA g^−1^). A combination of hydrothermal treatment and subsequent 3 h of heat treatment in air at 500 °C has used to fabricate freestanding Cr_2_O_3_ nanosheets/CC flexible anodes.^126^ The large surface area with better contact of active material with electrolyte improves the rate of Li-ion diffusion. Consequently, the hybrid anode delivered a high discharge capacity with good retention (1547.8 mA h g^−1^ at 0.1 A g^−1^ and retained 917.3 mA h g^−1^ after 200 cycles), and also demonstrated a specific capacity of 414.2 mA h g^−1^ after 400 cycles at 800 mA g^−1^. A freestanding Li_4_Ti_5_O_12_ cuboid arrays/carbon fiber cloth composite has been proposed as a high rate of LIBs anode materials, which are synthesized through the hydrothermal method, and subsequent lithiation ensuing thermal treatment at 800 °C.^127^ Subtle slits between nanocuboids improve the interlayer spacing and specific surface area as well as numerous micropores and mesopores of electrode synergistically improve the electrolyte penetration, Li^+^ transfer rate and shorten the diffusion path results in remarkable rate performance (169.1 mA h g^−1^ at 50C), and superior electrochemical stability (2.2% capacity loss at 10C after 1000 cycles). A flexible full cell was fabricated with using this anode and LiNi_0.5_Mn_1.5_O_4_/Al cathode, the cell delivered a reversible capacity of 109.1 mA h g^−1^ at 10C and maintained 100% capacity after 200 times of bending. V_2_O_5_ nanosheet arrays are grown on polydopamine decorated CC (mas loading of ∼2.1 mg cm^−2^) by solvothermal method then calcination at 350 °C for 2 h under air atmosphere,^128^ which exhibits a high rate capability (120 mA h g^−1^ at 4500 mA g^−1^), and excellent cycle performance (140 mA h g^−1^ at 600 mA g^−1^ after 100 cycles). N-doped carbon-coated V_2_O_5_ nanobelt arrays/CC (mass loading 1.2 mg cm^−2^) as high-performance flexible cathodes for LIBs has been fabricated *via* hydrothermal reaction after that thermal treatment.^129^ Additional N-doped carbon layer provides high conductivity and protects from direct exposure of active materials in the electrolyte. Besides, the nanobelt arrays formed a porous structure and the elastic properties of nanobelt arrays facilitate high ion diffusion rate and reduce pulverization simultaneously. Therefore, the cathodes demonstrated high rate performance (135 mA h g^−1^ at 2940 mA g^−1^), and good cycling stability (215 mA h g^−1^ at 147 mA g^−1^ after 50 cycles), which exhibit high capacity at low mass loading than previously mentioned V_2_O_5_ contained one. CoF_2_ NPs hydrothermally grown on conductive CC, which has been proposed as a conversion reaction cathode for flexible LIBs, it can be intact when bending with 180° angle.^130^ The composite cathode delivered a high capacity of 330 mA h g^−1^ at 100 mA g^−1^ after 200 cycles, and after 1000 cycles, a stable capacity of 100 mA h g^−1^ at a current density of 1 A g^−1^.

### 2.5. Carbon coating/foam-supported electrodes

#### 2.5.1. Carbon coating-supported electrodes

**Fig. 5:**
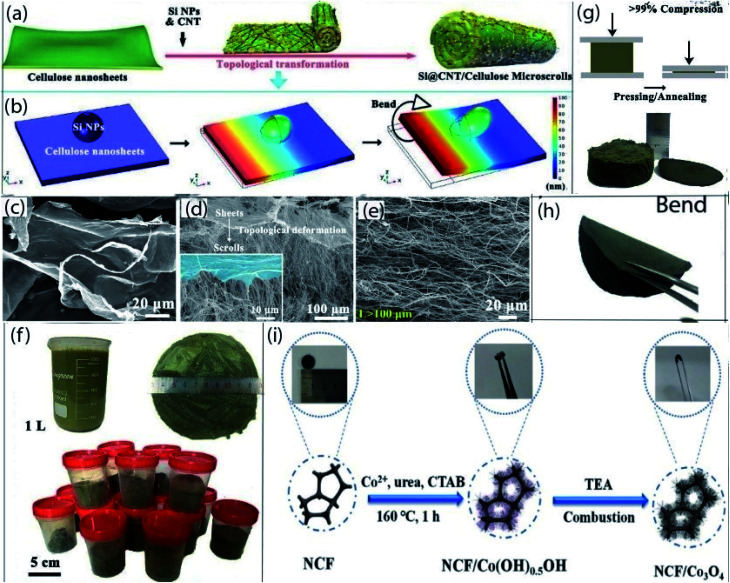
(a) Schematic illustration of the preparation process for Si@CNT/C-microscrolls, (b) simulation modeling of the topological deformation process of one side of a cellulose nanosheet with one Si NP, (c–e) SEM images of cellulose nanosheets, the transition stage that simultaneously shows the sheet and scroll structure, and a Si@CNT/C-microscroll, (f) digital photographs of 1 L of a Si@CNT/cellulose mixed solution along with the amount of Si@CNT/cellulose aerogel produced per batch, (g) free-standing Si@CNT/C-microscroll electrode obtained by pressing and annealing the aerogel (>99% compression), (h) bended state of Si@CNT/C-microscroll electrode. (a–h) Reprinted from ref. 134. (i) Schematic illustration of the synthesizing of binder-free N-doped carbon foam/Co_3_O_4_ electrode with photograph of flexible electrodes. (i) Reprinted from ref. 145.

In a carbon-coated integrated system, carbon provides electrical and mechanical supports. Recently, the amorphous carbon coating process comes into the view due to suitable electrical conductivity and good mechanical stability.^131–133^ Typically, thermal pyrolysis of carbon precursors on active materials is the widely applied technique to achieve carbon-coated integrated flexible electrodes (Fig. 5). Self-supporting flexible carbon micro scroll coated-Si@CNT (92% Si content) anodes for LIBs are prepared by using a combination of ultrasonication, freeze-drying followed by calcination at 800 °C for 2 h.^134^ In this architecture, surprising mass loading resolves the low active material loading issues. Moreover, high conductivity of carbon micro scroll and cage-like structure not only provided high conductivity but also prevent the pulverization of electrode as well as buffer the volume expansion of Si during the electrochemical reactions. Thus, the anode delivered a superior specific capacity (2700 mA h g^−1^ at 0.2 A g^−1^), long-term cycle life (above 2000 mA h g^−1^ after 300 cycles at 0.2 A g^−1^), and commercial level areal capacity (5.58 mA h cm^−2^). Hence, this novel production route improves the areal capacity and cycle life for high-performance LIBs. An N-doped carbon-coated/Li_4_Ti_5_O_12_ nanosheets self-standing films as high-performance LIB anodes are fabricated *via* a simple and scalable process including chemical lithiation, then thermal annealing followed by vacuum filtration.^135^ Highly transfer pathways for electrons and ions are provided by N-doped carbon and porous structures of nanosheets. So, the hybrid anode shows a capacity of (170 mA h g^−1^ at a current density of 175 mA g^−1^), superior rate performance (72% of theoretical capacity recovered at 17 500 mA g^−1^ over 100 cycles), and good cycle life (152 mA h g^−1^ after 100 cycles at 1750 mA g^−1^). Flexible paper-like Zn_2_GeO_4_ nanofibers anchored with amorphous carbon composites are prepared *via* dissolution and recrystallization method, which has been proposed as flexible anode materials for advanced LIBs.^136^ The superior electrochemical and mechanical performance of this electrode can be attributed to the synergistic effect of the flexible intertwined network, strong C–O–M (M = Zn, Ge) bonds, and highly conductive carbon anchored in the nanofibers skeleton. So, the anode exhibits excellent rate capability (373 mA h g^−1^ at 12 A g^−1^), and ultralong cycle life (∼820 mA h g^−1^ over 2000 cycles at 1 A g^−1^). N-doped carbon-coated LiTi_2_(PO_4_)_3_ as high-performance anode materials are fabricated through one-pot carbonization incorporating the sol–gel method, which has been reported for high areal capacity flexible aqueous LIBs.^137^ N-doped carbon coating provides high conductivity and helps to maintain structural integrity during electrochemical reactions and mechanical flexion. Thus, the anode exhibits a high capacity (114 mA h g^−1^ at 8C), superior rate capability (64 mA h g^−1^ at 20C), and long-term cycling durability (64.8 mA h g^−1^ at 10C after 2000 cycles). A novel ZnO quantum dots (ZnO-QDs) anchored in amorphous carbon multilayered sheet (ZnO-QDs@CMS) composite anodes are fabricated *via* low-temperature thermal treatment using pre-prepared zinc glycolate complex as a precursor.^138^ In this hybrid electrode, a flexible amorphous carbon network is beneficial for ensuring high conductivity, mitigating volume expansion, suppressing the aggregation of active particles, and favored the formation of fast LiZn alloy, which contributed to the high electrochemical performance as well as long-time stability of flexible anode. Consequently, the anode delivered a high reversible capacity (1015 mA h g^−1^ at 50 mA g^−1^ after 80 cycles), and long-term cycling stability (565 mA h g^−1^ over 350 cycles at 1000 mA g^−1^). Free-standing LiFePO_4_/carbon composite cathodes are prepared *via* a simple coating method by spreading out slurry onto a hydrophobic surface.^139^ The hybrid cathode shows a high initial capacity of ∼156 mA h g^−1^ at 0.1C and can be kept ∼133 mA h g^−1^ at 10C after 500 cycles.

#### 2.5.2. Carbon foam-supported electrodes

Carbon foams have been explored in LIBs as lightweight, highly conductive and flexible scaffolds for constructing pliable electrode materials.^140^ The porous structure of carbon foams is beneficial for containing active materials and rapid ion transportation. Several routes have been investigated such as template carbonization, bowing and carbonization, compression of exfoliated graphite, and assembly of graphene nanosheets, *etc.* for the preparation of carbon foams.^141^ This section will briefly present the recent development of carbon foam-based electrode materials for flexible LIBs (Fig. 5).

The self-knitted alpha-MnO_2_ fabric/carbon foam composite as flexible anode materials for LIBs are produced *via* using a hydrothermal process and subsequent CVD process.^142^ The porous structure of MnO_2_ fabric with highly conductive carbon foam, which greatly improves the electrons/ion transportation for electrochemical reactions and also maintained structural integrity during flexion. Thus, the anode delivered a high discharge capacity (840 mA h g^−1^ at 0.1 A g^−1^), excellent rate performance (86 mA h g^−1^ at 60 A g^−1^), and superior cycle life (450 mA h g^−1^ at 1200 mA g^−1^ after 1000 cycles). Moreover, a highly flexible was assembled by coupling this anode with LCO/Al cathode, the cell showed a high specific capacity of as high as 825 mA h g^−1^ at 200 mA g^−1^ and an impressive energy density of 2451 W h kg^−1^ at a power density of 4085 W h kg^−1^. Furthermore, a LED light still worked normally even when the battery bent, or rolled up continuously, demonstrated its promising potential use in highly flexible LIBs for foldable, stretchable, and wearable electronic devices. A binary metal sulfide, NiCo_2_S_4_ nanotube arrays are directly grown on flexible 3D N-doped carbon foam through a facile surfactant-assisted hydrothermal process and the subsequent sulfurization treatment, which has been proposed as flexible anode materials for high-performance LIBs.^143^ The superior electrochemical performance can be attributed to the unique material composition, integrated smart architecture by rationally designed hollow nanostructured, which facilitates fast electron/ion transfer, the large contact area with electrolyte as well as relaxing the strain-induced from lithiation/delithiation process. Consequently, the anode delivered a high reversible capacity and better capacity retention (1721 mA h g^−1^ at 500 mA g^−1^ and retained 1182 mA h g^−1^ after 100 cycles). Freestanding and flexible LIBs anodes composed of N-doped carbon foam/anatase TiO_2_ has been fabricated by using a combination of thermal pyrolysis, facile surface wetting, and subsequent hydrolysis process with a mass loading of as high as 5.31 mg cm^−2^, which is 2.5–4 times higher than that of other reported TiO_2_ based anodes.^144^ High conductivity and mesoporous structure of carbon foam network, the low volume change of nanocrystalline TiO_2_, good adhesion between carbon foam and active material, and high cut-off voltage helps to achieve high capacity (188 mA h g^−1^ at 200 mA g^−1^), and good cycle life (149 mA h g^−1^ after 100 cycles at 1000 mA g^−1^). Freestanding and flexible N-doped carbon foam/Co_3_O_4_ nanorods arrays (mass loading of ∼2.5 mg cm^−2^) as composite anodes are fabricated *via* using hydrothermal synthesis and unique triethylamine combustion in air method.^145^ The three-dimensional framework and porous structure of the composite anode facilitate high ion/electron transfer rate, improve contact area between the electrolyte and active particles, and suppress the pulverization of the electrode. As a result, the free-standing anode delivered a high discharge capacity of 672 mA h g^−1^ at 0.1 A g^−1^, and excellent cycling stability (576 mA h g^−1^ after 300 cycles at 1 A g^−1^). So, this is an effective way to prepared flexible and binder-free carbon foam-based electrode materials for LIBs.

### 2.6. Graphite-supported electrodes

**Fig. 6:**
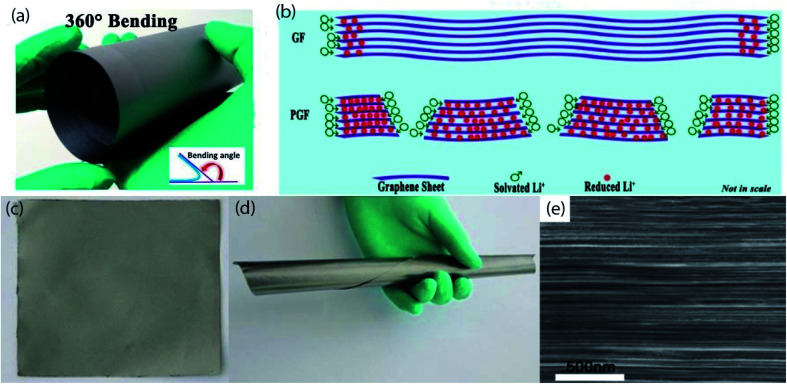
(a) Digital image of porous graphite film (PGF) being bent 360°, (b) a simple schematic illustration of the lithium intercalation into flexible graphite film and flexible PGF. Reprinted from ref. 151. (c) The image as prepared pyrolytic graphite film (PyGF), (d) rolled image of PyGF, (e) cross-sectional SEM image of PyGF. (c–e) Reprinted from ref. 149.

Graphite is one of the most widely used materials in the construction of LIB electrodes. Owing to high stability, stable electrochemical properties, good conductivity, impressive theoretical capacity, abundant in natural source, the yield capacity density can be closed to the theoretical specific capacity, and can be exhibit superior mechanical flexibility of graphite, results in graphite became an ideal anode active material as well as pliable conductive current collector/substrate for both of anode and cathode materials (Fig. 6).^146,147^ Such as a flexible anode composed of pyrolytic carbon-coated nano-sized Si particles/exfoliated graphite has been prepared through double step heat treatment processes.^148^ In this composite anode, porous structure with high conductivity of exfoliated graphite provides high electrons/ions transfer pathways. So, the anode delivered a high capacity of 902.8 mA h g^−1^ at 200 mA g^−1^ and retained 98.4% after 40 cycles. A Kapton-type polyimide film sandwiched between two polished graphite plates then carbonized at different temperature of 600°,1800°, and 2200 °C in Ar atmosphere, which has been reported as an anticorrosive flexible cathode current collectors in lithium bis(trifluoromethane sulfonyl)imide electrolyte system. Afterward, LiMn_2_O_4_ cathode active materials are grown on flexible graphite current collector.^149^ In addition, graphite film with active materials possess extreme flexibility that can be rolled up and could be altered into a variety of shapes as well as sizes enabling cell design flexibility. Moreover, the cathode exhibits a high discharge capacity (135 mA h g^−1^ at 0.2C), and outstanding cycle life (89% capacity retentions after 1000 cycles at 0.5C), and also retained 81% capacity over 300 cycles even when temperature raised up to 55 °C. On the contrary, in the case of the aluminum-based current collector, the LMO/Al cathode is completely contaminated after a few cycles. Hence, graphite film is more reliable to fabricate flexible electrodes for high safety LIBs. A novel additive-free approach, vertically aligned Li_4_Ti_5_O_12_ nanowire arrays are hydrothermally grown on flexible ultrathin free-standing CVD-grown graphite film, which has been proposed as flexible anode materials for LIBs.^150^ The high conductivity of graphite film and porous structure of LTO nanowire arrays synergistically facilitates fast electron/ion movement rates. As a result, the hybrid anode shows a high capacity (158 mA h g^−1^ at 0.5C), superior rate performance (104 mA h g^−1^ at 60C), and impressive cycling performance (110 mA h g^−1^ at 20C rate and improve 6% over 500 cycles). Another, graphite films (GF) are roll-to-roll synthesized *via* pyrolysis of polyimide films at horizontal-type ultra-high temperature graphitization furnace, then large pores are introduced in GF by directly drilled on flexible GF by laser drilling process, which can be directly served as flexible anode and cathode current collectors for LIBs.^151^ In addition, the pores of GF tremendously enhances the active sites for Li^+^ storage by providing high conductivity as well as help to tolerate mechanical deformation. Moreover, porous graphite film (PGF) anode exhibits an initial discharge capacity of 275 mA h g^−1^, which was increased to 359.2 mA h g^−1^ after 99 cycles at 15 mA g^−1^. On the other side, PGF/LiNi_0.5_Mn_1.5_O_4_ cathode possesses an initial specific discharge capacity of 127.5 mA h g^−1^ and retained 106.2 mA h g^−1^ after 400 cycles at 1C rate. Furthermore, both of these anode and cathode can recover its initial state even after 200 bending cycles with the largest bending angle of 180°. Inspired from the above results, high energy (5 V-class) flexible full cell has been assembled using that anode and cathode, the cell delivered an impressive discharge capacity of 90.8 mA h g^−1^ at 0.5C even after 400 cycles, and also lighted up four LEDs after several bending cycles. These results demonstrated the feasibility of PGF for the replacement of the Cu/Al current collectors. Hence, this low-cost and simple fabrication technique opens up the potentiality for the large-scale production of flexible electrodes for the building of LIBs for wearable/deformable electronics.

### 2.7. Important factors for developing carbonaceous materials based flexible electrodes

From the previously discussed topics, carbonaceous materials-based electrodes possess greater electrochemical and mechanical performances. However, herein we find out some important factors related to the hindering of mass applications of carbonaceous materials in flexible electrodes for FLIBs. This section will briefly summarize those factors and probable solutions for the future development are also provided.

#### 2.7.1. For CNT-supported electrodes

CNT-based electrodes have some limitations due to (i) high cost, (ii) intricated preparation process, (iii) detachment of active materials, (iv) aggregation of CNT, (v) insufficient electrochemical performance and (vi) limited flexion. Therefore, insertion of active materials in hollow CNT skeleton will help to suppress the detachment of active materials, effectively accommodate the volume expansion of active materials, and generating the uniform SEI layer. In addition, heteroatom doping (*i.e.* nitrogen, boron) in CNT structure is another route to improving the conductivity and flexibility. Moreover, defect rich CNT for instance splitting optimized amount of carbon atoms from CNT structure helps to enhancing ion transfer rate. Furthermore, lowering the radius of CNTs improve the toughness while shortening the length decreased the voltage hysteresis and surface resistance, which can improve the capacity of electrodes. Additionally, heating at optimized temperature,^22^ and washed with H_2_SO_4_ prior^152^ of CNT films are significantly improve the electrochemical and mechanical performances. This discussion will help to developing CNT-based high-performance flexible electrodes for FLIBs.

#### 2.7.2. ForCNFs-supported electrodes

CNFs-based electrodes suffer from some limitations such as (i) long synthesis process, (ii) poor interaction with active materials due to lacking of surface functional groups, (iii) low surface area causes poor conductivity and electrochemical performance. Therefore, introducing defects in CNFs structure such as heteroatom doping, etching and creating pores may help to improve flexibility and overall storage capacity. Additionally, embedding the active materials into the inner holes of CNFs are effectively reduce the fast capacity fading by suppressing the detachment and relaxing the volume changes of active materials. On the other hand, introducing CNT/graphene in CNFs based electrodes are significantly enhance the capacity and cycle stability.^153,154^ After all, it is necessary to the investigation of new CNFs structure with large surface areas and more functional groups to further the application of CNFs in flexible LIBs.

#### 2.7.3. For graphene-supported electrodes

This type of electrodes is (i) costly, (ii) weak interfacial bonding active materials, (iii) restacking of graphene sheets, (iv) impeding in ion movement along vertical direction, (v) presence of large amount (≥10%), (vi) unstable surface functional groups, (vii) insufficient in electrochemical and mechanical performances. Thus, using appropriate surfactants during electrode preparation helps to reduce the restacking of graphene sheets, complete abolishing of unstable surface functional groups can improve the coulombic efficiency. Moreover, pre-lithiation or pre-forming of artificial SEI on the electrode surface can mitigate the large irreversible capacity, though they are having not been yet available in industrial mass production. Furthermore, heteroatom doping in graphene skeleton also enhances the conductivity and active sites for Li storage. On the other side, combining CNT or CNFs in graphene-based electrodes, which effectively improves the capacity and cycle life of electrodes.^155^ However, improvement of contact interaction, volumetric capacity, and mechanical strength for desirable flexion cycles are crucial for further applications of graphene in flexible electrodes for commercial LIBs.

#### 2.7.4. For CFT-supported electrodes

CFT-based electrodes are facing some limitations as (i) complex synthesis procedure, (ii) low density, (iii) difficulty in installing desired amount of active materials, (iv) integration into wearable devices, (v) large mass (more than 10 mg cm^−2^) compared with copper foil (around 0.7 mg cm^−2^), (vi) low rate performance, (vii) poor adhesion with active materials. Owing to the high cost of the CVD technique, other promising coating procedures, for instance, screen printing, dip coating, and spinning should be used in industrial positions to large-scale manufacturing. Optimizing the number of carbon fibers in CFT textures may help to reducing the mass of CFT substrates and increasing in rate performance. Moreover, functionalizing the carbon fibers in CFT textures will increase the adhesion strength with active materials *via* generating chemical bonding. After all, exploring a novel strategy that includes scalability is more important for the multifarious applications and the commercialization of CFT based electrodes in flexible LIBs.

#### 2.7.5. For carbon coating/foam-supported electrodes

Due to (i) more electrolyte consumption, (ii) low electrical conductivity, (iii) inhibition of ion transfer rate, (iv) limiting the flexibility, (v) poor volumetric and specific capacity, (vi) thickening the electrodes, the carbon coating/foam-based electrodes are not well accepted. Investigating novel production route, optimizing the carbon content, purifying the carbon parts, heteroatom doping, and introducing pores in carbon skeletons will help to sustaining the further development in this field.

#### 2.7.6. For graphite-supported electrodes

These types of electrodes have some shortcomings as: (i) insufficient capacity in grams, (ii) easy to collapse layered structured after several subsequent cycles, (iii) lower purity and occurring more parasitic reactions, (iv) seriously limiting the deformability, (v) shortening the batteries life. Usually, binder is required, when graphite films are used as current collector/substrate. Therefore, new fabrication strategy and structural modification may solve the deformability related issues and integration of high capacitance active materials without binder can improve the capacity of electrodes. One the other hand, low-cost, environmental benignant and natural abundance advantages can support retaining its usefulness in the fabrication of flexible electrodes for LIBs.

### 2.8. Possible solutions for improving the mechanical sustainability of CM skeletons

In case of pliable electrodes, mechanical flexibility is the most crucial factor. To date, a lot of experimental works have been conducted to improve flexibility of electrodes without compromising energy density and capacity. Still this is a dilemma, how to achieve desirable flexion cycle without any lost in overall electrochemical performances of pliable electrodes? Limited flexion of conductive CMs, and detachment of active materials during flexion have been addressed as most responsible factors for this difficulty. In binder-free flexible electrode systems, CMs are mainly provide the mechanical support of deformable electrodes. Thus, it is important to enhance the mechanical properties of CM skeletons. In this section we will provide a brief understanding on how to improve the mechanical sustainability of pliable electrodes under long-term deformability.

Doping with appropriate hetero-atoms is a widely known strategy to improve mechanical and electrical performances of CMs. For instance, Khavrus *et al.* produced Boron-doped SWCNT by a post thermal diffusion method,^156^ Liu *et al.* reported N-doped CNT arrays,^157^ Yue *et al.*^158^ and Selvakumar *et al.*^159^ reported N-doped porous graphene films, Cao *et al.*^160^ reported a highly elastic N-doped carbon foam current collector that was synthesized *via* thermal pyrolysis of polyurethane foam. In addition, Zou *et al.*^161^ have reported boron and nitrogen co-doped holey graphene films, which possess improved electrical and mechanical performances than pristine graphene films. Therefore, doping with heteroatom will improve the mechanical performance of CMs supported flexible electrodes.

Guo *et al.*,^162^ and Xiong *et al.*^163^ have been synthesized highly flexible and porous graphene films with enhanced mechanical performance. In addition, Liu and co-workers^164^ have proposed a porous carbon film composed by CNT as a current collector for supercapacitor that exhibited high flexibility and ion diffusivity. On the other side, 3D porous networks of CMs have also attracted huge research attentions due to their long-term morphological structures, low electrical resistance rising, and better structural integrity under outer mechanical stress. For example, Ummethala *et al.*^165^ and Lee *et al.*^166^ have proposed a 3D interconnected and porous CNT foam type materials with significantly enhanced mechanical flexibility. Graphene based 3D interconnected porous nanostructures also possess higher mechanical performance.^167^ Ren *et al.*^168^ synthesized CNT and graphene contained 3D interconnected hybrid foam for LIBs application and they demonstrated that this hybrid material-based electrode shows only negligible amount of resistance increasing after several times of flexion. Further, Tang *et al.*^140^ have fabricated 3D interconnected and extremely flexible carbon foams with high conductivity (177 S cm^−1^), and porosity (99.6%). Thus, formation of porous and 3D interconnected network by CMs also increases the mechanical properties and will be sustained after long-term flexion.

In case of CNFs, doping is also widely studied strategy for the improving in mechanical and electrical properties.^169,170^ On the other hand, porosity engineering plays an important role to enhancing mechanical performance of CNFs by mitigating the outer stress.^79^ Individual CNT and graphene sheets prone to aggregation with less mechanical flexibility. In a review article, Zhang *et al.*^171^ summarized that CNT fibers exhibited intrinsic mechanical flexibility and electrical conductivity. Cheng *et al.*^172^ also summarized the properties of graphene fibers and they conclude that graphene fibers (GFs) have excellent mechanical and electrical performances though GFs still require massive improvement.

Cheng *et al.*^173^ reported CNT-graphene hybrid fiber-based textile for the supercapacitor applications, which exhibited excellent flexibility. Liu *et al.*^174^ also prepared CNT-graphene hybrid fibers based stretchable (200%) textiles. Hence, CNT and graphene fibers can be the better alternatives of conventional carbon fiber for the fabrication of CFT with high flexibility and electrochemical performance.

Jin *et al.*^175^ and Meng *et al.*^176^ synthesized poly(3,4-ethylenedioxythiophene) (PEDOT) coated CNT and graphene composite materials. Both of them exhibited excellent flexibility, while maintaining structural integrity and electrical conductivity after several times of flexion. Besides, this electrically conducting polymers (ECP) coating can be acted as protecting the CMs from the aggregation or detachment and reduce the overall resistance. As a result, coating with ECPs is an excellent route to improve flexibility without significant changes in resistance. On the other hand, as a binder ECPs are better than non-conducting polymers because of they can simultaneously enhance the flexibility and electrical conductivity of overall electrodes.

## 3. Metal mesh/fabric, nanowire, and foam/foil-supported flexible electrodes

### 3.1. Metal mesh/fabric-supported electrodes

**Fig. 7:**
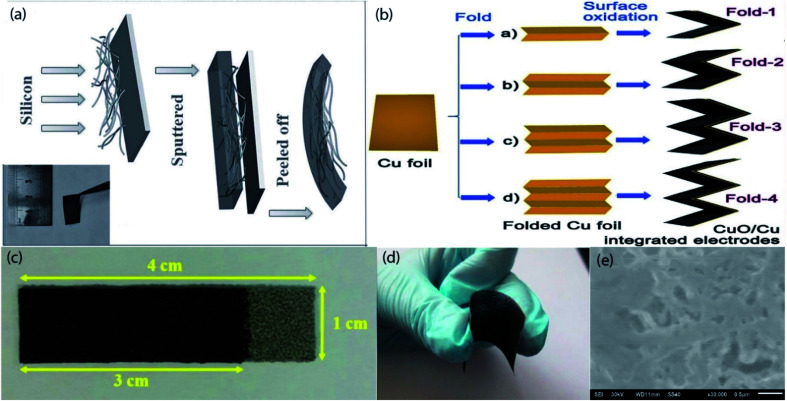
(a) Schematic image of magnetron sputtering method to deposit silicon. Reprinted from ref. ^184^. (b) Schematic illustration of fabrication procedures for four CuO/Cu integrated fold electrodes: One-fold, two folds, three folds and four folds. Reprinted from ref. ^193^. (c) Optical image of a positive electrode using the 3D structured NiCr alloy metal foam of the bendable Li secondary battery. Reprinted from ref. ^191^. (d) Photograph of as-prepared coaxial MnO_2_/CNTs nanocomposite freestanding membrane, (e) SEM image of the coaxial MnO_2_/CNTs nanocomposite freestanding membrane on SSM substrate anode after 200 cycles. (d and e) Reprinted from ref. ^179^.

Owing to low cost, high electrical conductivity, large surface area, favorable mechanical sustainability under structural deformations, considerable thickness, and smaller or no capacity contribution of metal mesh/fabric based current collector,^177^ emerged as a promising alternative of carbon cloth for developing cost-effective and flexible but effective current collector/substrate for flexible LIBs (Fig. 7). 3D stainless steel fibril mat (SFM)-supported silicon anode materials for high-performance flexible LIBs have been fabricated through a simple method using radio frequency (RF) magnetron sputtering technique.^178^ SFM created an interconnected mat-like configuration with open spaces between neighboring fibrils, results in fast ions and electrons transfer rate through the whole electrode. As a result, the anode delivered a high discharge capacity (∼3000 mA h g^−1^ at 300 mA g^−1^), excellent capacity retention (90% capacity retention at 2000 mA g^−1^ after 200 cycles). Moreover, using this anode a flexible full cell was assembled, the cell lost a little capacity (86% capacity retention at 1000 mA g^−1^) even after 150 times of bending. A freestanding coaxial MnO_2_/CNTs nanocomposite membrane grown on stainless steel mesh (SSM) *via* a two-step process: first, CNTs were grown on SSM by CVD process, then MnO_2_ active particles are coated on CNTs surface by electrodeposition, which can be worked as a flexible anode material for high-performance LIBs.^179^ SSM enables additional conductivity and porous structure facilitates rapid ion movement rates. Thus, the hybrid anode exhibits a high capacity and better cycling stability (2062 mA h g^−1^ at 200 mA g^−1^ and retained 80% capacity retained after 260 cycles), good rate performance (474 mA h g^−1^ at 1600 mA g^−1^). Self-standing porous LiCoO_2_ nanosheet arrays/Au-coated stainless-steel 3D cathodes has been prepared by a facile hydrothermal lithiation process followed by quick calcination in the air using Co_3_O_4_ nanosheet arrays as the precursor.^180^ Au–Co–O interface is formed during annealing, which is beneficial for enhancing adhesion strength between active material and substrate. Moreover, 3D configuration with the mesoporous structure of nanosheet facilitates electrolyte infiltration, relaxing volume expansion of LCO, and shortening the diffusion path of Li^+^ ions. Consequently, the composite cathode demonstrated outstanding rate capability (104.6 mA h g^−1^ at 10C), and long cycle life (81.8% of capacity retention after 1000 cycles at a current density of 0.1C). Hence, these impressive results exhibited its potentiality in the comprising of high-performance flexible LIBs. 3D textile-based cathodes (mass loading of 2.5 mg cm^−2^) are synthesized by combining V_2_O_5_ hollow multishelled structures (HoMSs) with conductive Ni/cotton fabric through a sequential templating approach for high-performance flexible LIBs.^181^ V_2_O_5_ HoMSs provides high surface-to-volume ratio and more active sites for Li^+^-ion storage and also effectively buffer the stress from volume changing during cycling. On the other side, metal fabric with a loose texture facilitates high electrical conductivity with a large amount of electrolyte infiltration simultaneously. Thus, the cathode retains a very high capacity of 224.2 mA h g^−1^ even after 500 subsequent cycles at a current density of 100 mA g^−1^. Moreover, under a large bending angle of 180°, the voltage remains barely changed after hundreds bending and folding cycles as well as no obvious resistance changes after 1000 bending cycles. Consequently, this unique investigation can be an open an extended avenue for the fabrication of flexible LIBs.

### 3.2. Metal nanowires-supported electrodes

Flexible current collectors have been fabricated by using metal nanowires (MNWs), enhances electrolyte penetrability and the diffusion kinetics of electron and ions, which led to improving rate performance. Owing to the high surface area, metallic electrical conductivity, and suitable mechanical ductility of metal nanowires (MNWs) have been extensively studied as flexible current collectors building element.^182,183^ Based on these useful properties, MNWs can be a potential alternative of carbon nanomaterials based on current collectors. Typically, filtration of MNWs contained dispersions has been used to fabricate flexible current collectors, later active materials are coated or deposited on as formed current collectors; or, one-step filtration of the dispersions of active materials and MNWs have also been used to fabricating MNWs-based flexible electrodes for LIBs. To date, several research groups have been extensively studied the MNWs based flexible electrodes in LIBs (Fig. 7). For example, a Cu NWs current collector sheets has been prepared by vacuum filtration, then Si active particles were deposited on Cu NWs current collector *via* radio frequency magnetron sputtering method, which can be act as a flexible anode for LIBs.^184^ Cu NWs sheet served as a flexible and highly conductive current collector and also enhances the electron transport kinetics with providing the stability of the composite anodes. As a result, the hybrid anode delivered a high discharge capacity with good capacity retention (3850 mA h g^−1^ at 0.84 A g^−1^ and retained 89.4% after 60 cycles), which is almost twice that of Si@Cu foil and Si@Ni-foam-based anodes. A composite anode composed of copper nanowires (Cu NWs)/MWCNT for fast charging/discharging LIBs are produced *via* simple filtration followed by sintered at 180 °C for 30 min in glycerol reducing conditions.^185^ The flexible anode with a mass ratio of 7 : 1 (Cu NWs : MWCNT) shows good electrochemical performance, at the rate of 5C the anode maintained 89.75% of capacity of 0.5C rate. Moreover, using this anode a flexible LIB retained more than 92.8% of its capacity after 1000 cycles under a continuous bending with a bending radius of 10 mm. Furthermore, using this anode a constructed LIB can be 55.78% charged at a 5C rate only in 12 min. Therefore, this is an effective route to comprise ultrafast charging flexible LIBs for next-generation advanced electronic devices. Binder-free, all inorganic nanowire bilayer (Cu/Ge) mesh as flexible anodes for high-performance LIBs has been fabricated by a step-by-step process; first, the dispersion of Cu NWs are drop cast on polytetrafluoroethylene (PTFE) mold, then drying, subsequently dispersion of Ge NWs drop cast on as obtained Cu NWs layer followed by calcination at 500 °C under Ar/H_2_ atmosphere for 2 h.^186^ High conductivity with enormous open spaces between adjacent NWs is beneficial for rapid ion/electrons transfer. As a result, the hybrid anode exhibits a high specific capacity (1153 mA h g^−1^ at 0.1 A g^−1^), excellent rate capability (359 mA h g^−1^ at 20 A g^−1^), long-term cycling stability (1000 cycles and 1300 cycles when operated at 1 A g^−1^ and 10 A g^−1^), and large areal capacity (4.13 mA h cm^−2^ after 50 cycles). Moreover, the volumetric capacity of Ge/Cu mesh anodes are 10 times enhanced than other Ge electrodes combined with graphene and CNFs.

### 3.3. Metal foam/foil-supported electrodes

Metal foams are usually explored as a 3D porous current collector or as flexible conductive scaffolds. Owing to unique porous architecture, active materials slurries can be embedded into the inner pore spaces led to suppress the volume expansions of active particles as well as curb the disengagement of active materials during the electrochemical reactions or mechanical deformation, offers the high surface area for redox reactions, shortening the diffusion path of Li^+^ ion results in greatly improve the overall capacity of electrodes.^187^,^188^ Besides, metal foams, metal foils are also investigated as a highly conductive and flexible current collectors for the building of pliable electrodes.^189^ Generally, coating or depositing of active materials onto the metal foam/foils have been employed for the preparation of pliable electrodes (Fig. 7). So far, various types of metal foam/foil based flexible electrodes have been explored by different research groups. This section will comprehensively introduce the recent development on metal foam/foil based pliable electrodes for LIBs. For instance, a 3D flexible Cu–Sn current collectors has been synthesized by depositing metals on polyurethane (PU) foam followed by removal of PU by heat treatment. Afterward, as-obtained Cu–Sn metal foams are calcined at 500 °C for 8 h under vacuum. Finally, LFP slurries are filled in the pore spaces of metal foam to yield flexible LiFePO_4_/Cu–Sn cathodes for LIBs.^190^ In this system, 3D porous and conductive current collector facilitates high ion and electron transfer rates, and additional heat treatment is beneficial for resisting the rapid capacity fading. As a result, the hybrid cathodes delivered a superior capacity of above 400 mA h g^−1^ after 50 cycles at a current density of 100 mA g^−1^. Three-dimensional NiCr-alloy metal foams are fabricated by the step-by-step process; first, Ni foams are produced by PU templating process, then NiCr alloy powder adsorbed on Ni foam followed by annealing to obtain NiCr-alloy foam. Finally, LiFePO_4_/C active material slurries deposited in the pores of foam and then reheated at 130 °C for 8 h under vacuum.^191^ High electrochemical performance and mechanical pliability could be attributed to the 3D porous foam type conductive current collector using this flexible LiFePO_4_/C@NiCr cathodes a pouch type battery exhibits a superior electrochemical performance (3.5 mA cm^−2^ at 0.2C), good capacity retention (94% capacity retained after 50 cycles), and excellent mechanical performance (3.08 mA h cm^−2^ capacity maintained even after 200 times bending at 0.2C with bending radii of 12.5 and 2.5 mm). Three-dimensional web-like anode materials are fabricated through numerous Li_4_Ti_5_O_12_ nanowires directly grown on Ti foil *via* a facile template-free route, which has been proposed as ultralong-life and high-rate anodes for high-performance flexible LIBs.^192^ In this 3D configuration, 1D nanowires significantly enhance the active sites for Li storage, shorten the transport length of electrons and ions, and helps to improve the contact area between active material and electrolyte. So, the anode shows a superior rate capability (103 mA h g^−1^ at 80C), ultralong cycle life (153 mA h g^−1^ and 115 mA h g^−1^ after 5000 cycles at 2C and 20C, respectively), and only slight capacity decay in every single bending cycle. To produce high areal capacity anodes for flexible LIBs, CuO nanorods are grown on folded Cu foils through surface oxidation reaction.^193^ In this folding configuration, every fold adds one more Cu layer than the fold number. Such as one-fold and creates two Cu layers (fold-1), twofold and created three Cu layer (fold-2), and so on. This unique fabrication technique can not only enable the high adhesion between CuO NRs and Cu foils but also help to loads more CuO NRs. Consequently, CuO NRs/five-layers Cu foil (fold-4) achieved a outstanding areal capacity of 13.95 mA h cm^−2^. In addition, fold- 3 anodes maintained a high reversible areal capacity of 5.32 mA cm^−2^ after 100 cycles. On the other side, no observable cracks are found after 100 bending cycles in this integrated electrode systems. Thus, a flexible full cell comprised with a fold-3 anode and LiMnSiO_4_/C textile cathode, the cell exhibits an aerial capacity of 2.7 mA h cm^−2^ at a current density of 0.3 mA cm^−2^ with no significant capacity decay after 100 bending cycles. So, this unique fabrication technique can be an effective route to designing high areal capacity integrated anode materials with favorable deformability for LIBs.

### 3.4. Possible solutions for developing metals-supported stable flexible electrodes

As metallic current collectors prone to corrosion during long-term cycling, elevating the resistance, and crack formation in the stressed zone while repeatedly mechanical deformation leads to rapid structural failure. In order to overcome such shortcomings, researchers have provided several types of suggestions such as, coated with graphene,^194–196^ growing of CNTs on metallic current collectors^197–199^ surface modifying with conducting polymer,^200^ coated with carbon layers,^201,202^ surface modification^203^ In addition, Park *et al.* synthesized honey comb like 3D structured Al and Cu by using reactive-ion etching (RIE) process, which reduce the total mass of electrodes and improved the adhesion strength with active materials results in enhanced flexibility.^204^ Metallic fibril mesh-like configuration is another approach to preparing free-standing electrodes, which generates 3D conductive pathways through the whole electrodes and reduce the mass of electrodes while improve the capacity and flexibility.^178^ Moreover, 3D fibrous current collectors have low value of tortuosity and controlled porosity results in sufficient electrolyte diffusion and enhanced volumetric capacity.^205^ On the other hand, 3D fibrous type current collectors generate more adhesion towards active materials, increase the surface area, and lowering the total mass of electrodes. So, based on those aspects, 3D fibrous type metallic current collectors are more promising than foam based current collectors. Besides, joint-to-joint welding of metal NWs will improve the mechanical integrity of conductive metals parts in flexible electrodes under outer deformation stress.^206^

In summary, coating with above-mentioned materials are the effective strategy to enhancing electrical, and mechanical properties of metallic current collectors. Also, protect from direct exposure in electrolyte solutions that prevent the corrosion of metal parts which leads to improving electrochemical stability. On the other side, surface modification or designing 3D hierarchical structure enhancing the adhesion strength with active materials also leads to enhancing cycling stability and overall capacity of electrodes. Therefore, (i) coated with conductive polymers/carbonaceous materials and active materials combining with optimized amount of binders on to the rough surface of 3D hierarchical metallic current collectors; or (ii) using the joint-to-joint welded structured metallic mesh/3D fibrous type current collectors for the preparation of flexible electrodes will help to sustaining the future metals supported pliable electrodes.

## 4. Understanding of chemical and configurational stability

Electrochemical performance and cycle life of FLIBs predominantly depends on the stability of electrodes integrity during electrochemical reactions and mechanical deformations. Metal-based current collectors are most popular due to their high conductivity, easy processability, low price, and commercial availability.^10,207^ On the other side, over the past decade, carbonaceous materials are also extensively studied in deformable electrodes owing to their enough electrical conductivity, desirable electrochemical, and mechanical performances.^8^ On contrary, high cost intricated synthesis procedures and lacking of industrial production routes of carbonaceous materials limiting their mass applications. However, with respect to carbonaceous materials, metal-based flexible electrodes suffer from various serious issues that generate instability of metal-supported flexible electrodes. Such as corrosion of metallic current collectors through oxidation/reduction reactions,^208,209^ poor interfacial contact with active materials,^16^ high interfacial resistance,^210^ Al and stainless steel based current collectors are completely decayed at voltages over 4.2–4.5 V *vs.* Li^+^/Li^208,211,212^ occupying huge segments of total masses of anodes and cathodes respectively without any contributing in capacity, fast detachment of active materials during deforming, accelerating crack formation under repeated mechanical flexion, and using a large amount of binder. In addition, Wang *et al.* reported that the contact angle of a graphite–CNT interface (19°) is smaller than graphite–Cu interface (48°) results in low adhesion between graphite and Cu, which causes the fast detachment of graphite from Cu current collector.^16^ Cui *et al.* reported that with respect to pyrolytic graphite film, Al current collector rapidly contaminated in lithium bis(trifluoromethane sulfonyl)imide electrolyte containing system when temperature rises to 55 °C. Also, PGF based electrode possesses a greater degree of flexibility than that of Al-based electrode.^149^ From the abovementioned discussion, we can assume that carbonaceous materials are highly reliable for the fabrication of pliable and stable electrodes for FLIBs.

## 5. Techniques for mechanical deformation evaluation

Flexibility is the crucial event for the implementation of pliable electrodes in deformable LIBs. Up to date, several methods have been proposed to evaluate the degree of flexibility and failure modes but still lacks available established evaluation method and standard degree of flexion limit for the measurement of the flexibility of pliable electrodes. However, in this part, we have tried to briefly highlight the key parameters and useful methods for the measurement of the flexibility of the pliable electrodes. For instance, end-to-end distance, bending angle, and bending radius are important parameters for the evaluation of the bending status of bendable electrodes.^213^ Firstly, end-to-end (*L*) distance means that the distance between the two ends of flexible electrodes obtained by the bending of the electrodes.^214^ Secondly, the bending angle (*θ*) expresses the rotating state of the moving end of an electrode.^215^ Though *L* and *θ* provide the general understanding of bending conditions but in the case of long electrodes, it is hard to understand the actual bending status. Besides, these two parameters also failed to provide a general concept of the failure model of flexible electrodes. Bending radius (*R*) has been proposed as another parameter to measure the actual bending status of flexible electrodes.^216^ However, various techniques have been suggested to measure the actual *R* of flexible devices such as the constant cylinder method, collapsing radius geometry process, and *X*–*Y*–*θ* method. In the constant cylinder method, flexible electrodes are manually wrapped around a cylinder with a known diameter then measure the *R* of flexible electrodes.^217^ Nevertheless, the non-uniform stress distribution is observed due to manual handling. Also, this process is not appropriate for the measurement of perfect *R* and failure strain of flexible electrodes for the continuous bending process. In the case of collapsing radius geometry measurement, testing devices are placed between the two parallel positioned squeezable plates. The *R* was obtained as half of the two ends of testing devices.^218^ though this process can be applicable for the continuous process but non-uniform stress distribution and shape of the curved structure is not perfect cylindrical. Consequently, this method is not appropriate for the measurement of the real value of *R*. In the case of *X*–*Y*–*θ* method, the testing sample was initially placed horizontally in a testing device, the testing device consists with one fixed plate and one moving plate. During test process, moving end form an arc-shaped condition with fixed end of testing sample, where the speed and direction of moving end was controlled by rotary motorized actuators.^219^ In this process, *X* refers to initial horizontal direction, and *θ* refers to the angle between *X* and the tangent of the moving end. The bending angle is controlled between 0° to 180° and the smallest *R* is obtained as less than *L*_0_/π (*L*_0_ defined the length between the two ends of testing sample). Thus, this process can be used for the measurement of *R* due to the bending condition is controlled by an automated machine with uniform distribution of stress and variation speed of bending angle that can be extended for the estimating *R* of the flexible electrodes. On the other side, when the active materials distributed ununiformly on conductive supports/substrates then these aforementioned methods cannot predict the actual *R*. So, to calculate the actual bending radius of the non-homogeneously distributed active materials contained flexible electrodes, Island film method have been proposed.^220,221^ Further, Stoney's formula suggested method to find out the films thickness and residual stress concerning bending radius.^220,222^

Therefore, from the abovementioned discussion, we can assume that determination of bending radius is more preferable than any other parameters to describe the bending state of the flexible electrodes. In addition, among various methods, *X*–*Y*–*θ* method is highly recommended to calculate the bending radius. Further, island film and Stoney's theory should be used to attaining more realistic values of bending radius.

## 6. Comparative study on electrochemical performances

**Fig. 8:**
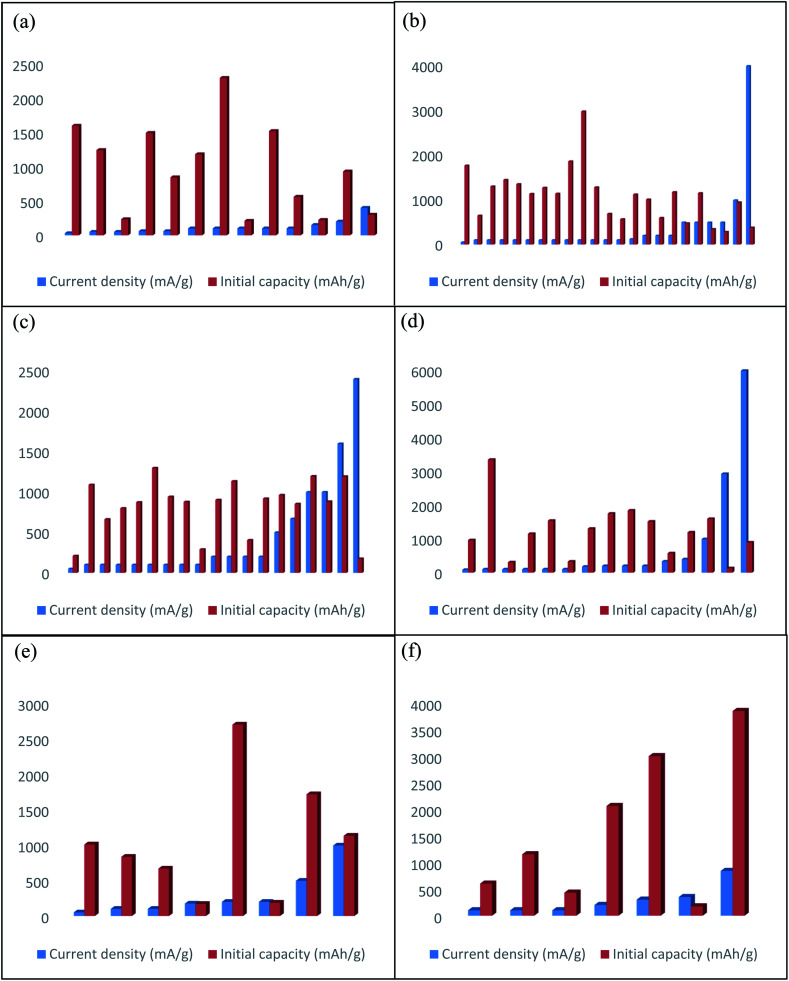
Highest initial capacity at the certain current density of (a) CNT,^24–26,30,31,33,36,39,40,42–44,48^ (b) CNFs,^52,55–60,62–75,77,78^ (c) graphene,^85–87,90–102,104,105,107^ (d) CFT,^111,113–120,122,123,125,126,129,130,134–136,138,142–145^ (e) carbon coating/foam,^134–136,138,142–145^ and (f) metals-supported^178,179,181,184,190,192^ flexible electrodes.

Applications of flexible electrodes in FLIBs are mainly dependent on their stability of electrochemical performance. Among various types of electrode materials CMs and metals-based electrodes exhibit greater performance due to their high electrical conductivity and low internal resistance. This part will provide a comparative understanding on their overall electrochemical behaviors based on our reported works in CMs and metals-supported flexible electrodes sections. Firstly, in Fig. 8 we have presented the initial capacities of CMs and metals-based electrodes, and in Fig. 10a we systematically estimate the average initial capacities at the average initial current densities by using simple mathematical average calculation method. Moreover, Table 4 contained all of the initial and final performances of every type of electrodes that are depicted in subsequent graphs. Thus, from Fig. 10a CMs such as CNT, CNFs, graphene, CFT, and carbon coating/foam-based electrodes exhibits the average initial capacities of 972.71, 1092.32, 825.55, 1235.97, and 1055.43 mA h g^−1^ at the average current density 155.38, 390.13, 464.21, 802.66, and 290.62 mA g^−1^ respectively while metal-based electrodes demonstrated an average initial capacity of 1610.25 mA h g^−1^ at the average current density of 284.28 mA g^−1^. Owing to the higher electrical conductivity of metals than that of CMs, which stimulates greater average initial electrochemical performance?

**Fig. 9:**
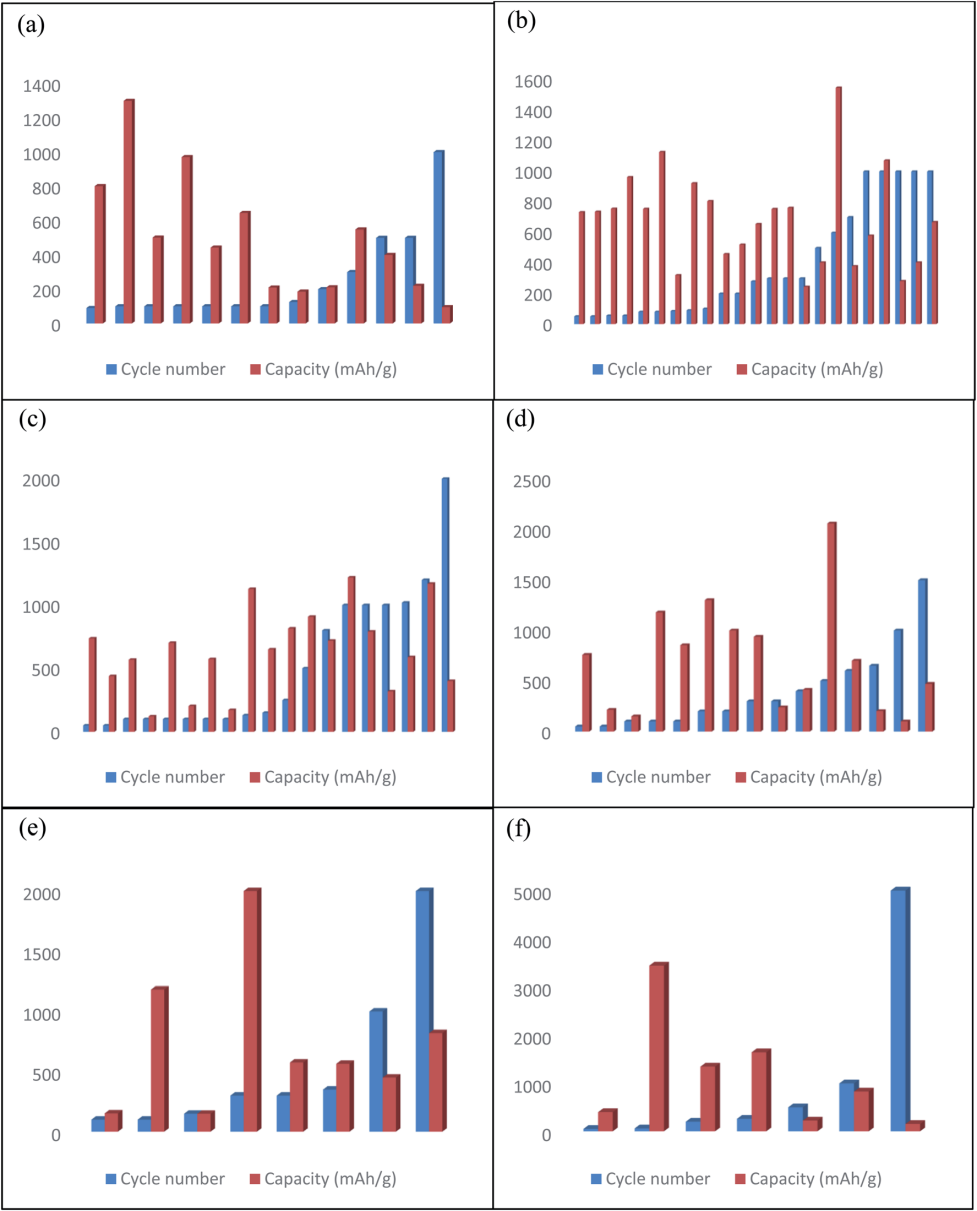
Capacities after certain number of electrochemical cycles of (a) CNT, (b) CNFs, (c) graphene, (d) CFT, (e) carbon coating/foam, and (f) metals supported flexible electrodes (these data also collected from the same sources as presented in Fig. 8).

**Fig. 10:**
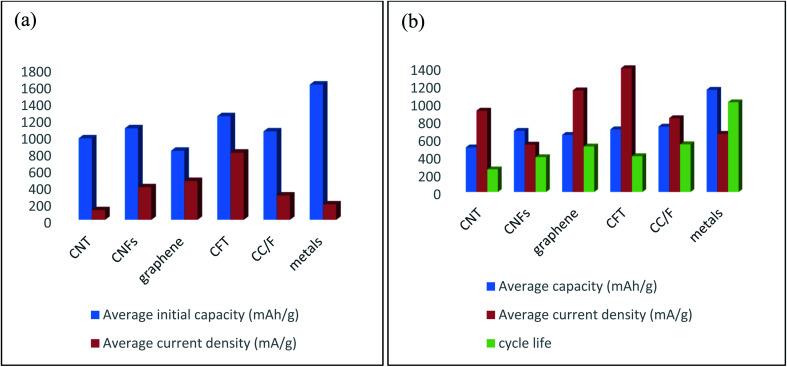
(a) Average initial capacities at the average current densities, and (b) average capacities, average current densities, and average cycle life of all types of electrodes (these data also collected from the same sources as reported in Fig. 8).

**Table 4:** Initial and final performances of all types of electrodes

Initial performance (ref.) [capacity/current rate/cycle life]					
CNT-supported electrodes	CNFs-supported electrodes	Graphene-supported electrodes	CFT-supported electrodes	CC/F-supported electrodes	Metals-supported electrodes
1780 mA h g^−1^/50 mA g^−1^ (ref. 52) [1550 mA h g^−1^/500 mA g^−1^/600]	1780 mA h g^−1^/50 mA g^−1^ (ref. 52) [1550 mA h g^−1^/500 mA g^−1^/600]	1198 mA h g^−1^/1000 mA g^−1^ (ref. 85) [1170 mA h g^−1^/1000 mA g^−1^/1200]	3362 mA h g^−1^/100 mA g^−1^ (ref. 111) [2061 mA h g^−1^/1000 mA g^−1^/500]	2700 mA h g^−1^/200 mA g^−1^ (ref. 134) [2000 mA h g^−1^/200 mA g^−1^/300]	3000 mA h g^−1^/300 mA g^−1^ (ref. 178) [1350 mA h g^−1^/2000 mA g^−1^/200]
654 mA h g^−1^/100 mA g^−1^ (ref. 55) [756 mA h g^−1^/100 mA g^−1^/55]	654 mA h g^−1^/100 mA g^−1^ (ref. 55) [756 mA h g^−1^/100 mA g^−1^/55]	175 mA h g^−1^/2400 mA g^−1^ (ref. 86) [588 mA h g^−1^/1600 mA g^−1^/1020]	1761 mA h g^−1^/200 mA g^−1^ (ref. 113) [938 mA h g^−1^/200 mA g^−1^/300]	170 mA h g^−1^/175 mA g^−1^ (ref. 135) [152 mA h g^−1^/1750 mA g^−1^/100]	2062 mA h g^−1^/200 mA g^−1^ (ref. 179) [1649 mA h g^−1^/200 mA g^−1^/260]
287.4 mA h g^−1^/500 mA g^−1^ (ref. 56) [380.1 mA h g^−1^/200 mA g^−1^/700]	287.4 mA h g^−1^/500 mA g^−1^ (ref. 56) [380.1 mA h g^−1^/200 mA g^−1^/700]	1194 mA h g^−1^/1600 mA g^−1^ (ref. 87) [1220 mA h g^−1^/1600 mA g^−1^/1000]	968.6 mA h g^−1^/850 mA g^−1^ (ref. 114) [471.2 mA h g^−1^/900 mA g^−1^/1500]	1137.5 mA h g^−1^/1000 mA g^−1^ (ref. 136) [820 mA h g^−1^/1000 mA g^−1^/2000]	601.8 mA h g^−1^/100 mA g^−1^ (ref. 181) [224.2 mA h g^−1^/100 mA g^−1^/500]
1313 mA h g^−1^/100 mA g^−1^ (ref. 57) [581 mA h g^−1^/1000 mA g^−1^/1000]	1313/100 mA g^−1^ (ref. 57) [581 mA h g^−1^/1000 mA g^−1^/1000]	1091 mA h g^−1^/100 mA g^−1^ (ref. 90) [568 mA h g^−1^/100 mA g^−1^/100]	310 mA h g^−1^/100 mA g^−1^ (ref. 115) [150 mA h g^−1^/500 mA g^−1^/100]	1015 mA h g^−1^/50 mA g^−1^ (ref. 138) [565 mA h g^−1^/1000 mA g^−1^/350]	3850 mA h g^−1^/840 mA g^−1^ (ref. 184) [3441.9 mA h g^−1^/840 mA g^−1^/60]
1460 mA h g^−1^/100 mA g^−1^ (ref. 58) [1072 mA h g^−1^/1000 mA g^−1^/1000]	1460 mA h g^−1^/100 mA g^−1^ (ref. 58) [1072 mA h g^−1^/1000 mA g^−1^/1000]	904 mA h g^−1^/200 mA g^−1^ (ref. 91) [650 mA h g^−1^/200 mA g^−1^/150]	579 mA h g^−1^/335 mA g^−1^ (ref. 116) [203 mA h g^−1^/3350 mA g^−1^/650]	840 mA h g^−1^/100 mA g^−1^ (ref. 142) [450 mA h g^−1^/1200 mA g^−1^/1000]	1153 mA h g^−1^/100 mA g^−1^ (ref. 186) [830 mA h g^−1^/1000 mA g^−1^/1000]
1363.6 mA h g^−1^/100 mA g^−1^ (ref. 59) [655 mA h g^−1^/500 mA g^−1^/280]	1363.6 mA h g^−1^/100 mA g^−1^ (ref. 59) [655 mA h g^−1^/500 mA g^−1^/280]	854.3 mA h g^−1^/670 mA g^−1^ (ref. 92) [736.8 mA h g^−1^/67 mA g^−1^/50]	1603 mA h g^−1^/1000 mA g^−1^ (ref. 117) [700.3 mA h g^−1^/5000 mA g^−1^/600]	1721 mA h g^−1^/500 mA g^−1^ (ref. 143) [1182 mA h g^−1^/500 mA g^−1^/100]	432 mA h g^−1^/100 mA g^−1^ (ref. 190) [400 mA h g^−1^/100 mA g^−1^/50]
1131 mA h g^−1^/123 mA g^−1^ (ref. 60) [923 mA h g^−1^/123 mA g^−1^/90]	1131 mA h g^−1^/123 mA g^−1^ (ref. 60) [923 mA h g^−1^/123 mA g^−1^/90]	1135.2 mA h g^−1^/200 mA g^−1^ (ref. 93) [1129.2 mA h g^−1^/200 mA g^−1^/130]	1310 mA h g^−1^/180 mA g^−1^ (ref. 118) [1180 mA h g^−1^/180 mA g^−1^/100]	188 mA h g^−1^/200 mA g^−1^ (ref. 144) [149 mA h g^−1^/1000 mA g^−1^/149]	350 mA h g^−1^/173 mA g^−1^ (ref. 192) [153 mA h g^−1^/350 mA g^−1^/5000]
1145 mA h g^−1^/100 mA g^−1^ (ref. 62) [733 mA h g^−1^/100 mA g^−1^/50]	1145 mA h g^−1^/100 mA g^−1^ (ref. 62) [733 mA h g^−1^/100 mA g^−1^/50]	405 mA h g^−1^/200 mA g^−1^ (ref. 94) [122 mA h g^−1^/2000 mA g^−1^/100]	1851.9 mA h g^−1^/200 mA g^−1^ (ref. 119) [1302.3 mA h g^−1^/200 mA g^−1^/200]	672 mA h g^−1^/100 mA g^−1^ (ref. 145) [576 mA h g^−1^/1000 mA g^−1^/300]	
1018 mA h g^−1^/200 mA g^−1^ (ref. 63) [460 mA h g^−1^/200 mA g^−1^/200]	1018 mA h g^−1^/200 mA g^−1^ (ref. 63) [460 mA h g^−1^/200 mA g^−1^/200]	963.9 mA h g^−1^/500 mA g^−1^ (ref. 95) [908.7 mA h g^−1^/500 mA g^−1^/500]	1524 mA h g^−1^/200 mA g^−1^ (ref. 120) [854 mA h g^−1^/500 mA g^−1^/100]		
1284 mA h g^−1^/100 mA g^−1^ (ref. 64) [963 mA h g^−1^/100 mA g^−1^/55]	1284 mA h g^−1^/100 mA g^−1^ (ref. 64) [963 mA h g^−1^/100 mA g^−1^/55]	663 mA h g^−1^/100 mA g^−1^ (ref. 96) [438.5 mA h g^−1^/100 mA g^−1^/50]	900 mA h g^−1^/6000 mA g^−1^ (ref. 122) [240 mA h g^−1^/6000 mA g^−1^/300]		
954 mA h g^−1^/1000 mA g^−1^ (ref. 65) [736 mA h g^−1^/50 mA g^−1^/50]	954 mA h g^−1^/1000 mA g^−1^ (ref. 65) [736 mA h g^−1^/50 mA g^−1^/50]	800 mA h g^−1^/100 mA g^−1^ (ref. 97) [702 mA h g^−1^/100 mA g^−1^/100]	1200 mA h g^−1^/400 mA g^−1^ (ref. 123) [1000 mA h g^−1^/400 mA g^−1^/200]		
1149.4 mA hg ^−1^/100 mA g^−1^ (ref. 66) [805.8 mA h g^−1^/100 mA g^−1^/100]	1149.4 mA h g^−1^/100 mA g^−1^ (ref. 66) [805.8 mA h g^−1^/100 mA g^−1^/100]	210 mA h g^−1^/50 mA g^−1^ (ref. 98) [203 mA h g^−1^/50 mA g^−1^/100]	1156.5 mA h g^−1^/100 mA g^−1^ (ref. 125) [758.1 mA h g^−1^/700 mA g^−1^/50]		
1872 mA h g^−1^/100 mA g^−1^ (ref. 67) [755 mA h g^−1^/100 mA g^−1^/80]	1872 mA h g^−1^/100 mA g^−1^ (ref. 67) [755 mA h g^−1^/100 mA g^−1^/80]	874 mA h g^−1^/100 mA g^−1^ (ref. 99) [791 mA h g^−1^/1000 mA g^−1^/1000]	1547.8 mA h g^−1^/100 mA g^−1^ (ref. 126) [414.2 mA h g^−1^/800 mA g^−1^/400]		
481 mA h g^−1^/500 mA g^−1^ (ref. 68) [321 mA h g^−1^/500 mA g^−1^/85]	481 mA h g^−1^/500 mA g^−1^ (ref. 68) [321 mA h g^−1^/500 mA g^−1^/85]	1300 mA h g^−1^/100 mA g^−1^ (ref. 100) [320 mA h g^−1^/5000 mA g^−1^/1000]	135 mA h g^−1^/2940 mA g^−1^ (ref. 129) [215 mA h g^−1^/147 mA g^−1^/50]		
2987.7 mA h g^−1^/100 mA g^−1^ (ref. 69) [1128 mA h g^−1^/100 mA g^−1^/80]	2987.7 mA h g^−1^/100 mA g^−1^ (ref. 69) [1128 mA h g^−1^/100 mA g^−1^/80]	943 mA h g^−1^/100 mA g^−1^ (ref. 101) [573 mA h g^−1^/100 mA g^−1^/100]	330 mA h g^−1^/100 mA g^−1^ (ref. 130) [100 mA h g^−1^/1000 mA g^−1^/1000]		
1292.9 mA h g^−1^/100 mA g^−1^ (ref. 70) [754 mA h g^−1^/1000 mA g^−1^/300]	1292.9 mA h g^−1^/100 mA g^−1^ (ref. 70) [754 mA h g^−1^/1000 mA g^−1^/300]	920 mA h g^−1^/200 mA g^−1^ (ref. 102) [719 mA h g^−1^/2000 mA g^−1^/800]			
384 mA h g^−1^/4000 mA g^−1^ (ref. 71) [405 mA h g^−1^/1000 mA g^−1^/500]	384 mA h g^−1^/4000 mA g^−1^ (ref. 71) [405 mA h g^−1^/1000 mA g^−1^/500]	880 mA h g^−1^/100 mA g^−1^ (ref. 104) [400 mA h g^−1^/5000 mA g^−1^/2000]			
1161 mA h g^−1^/500 mA g^−1^ (ref. 72) [762 mA h g^−1^/500 mA g^−1^/300]	1161 mA h g^−1^/500 mA g^−1^ (ref. 72) [762 mA h g^−1^/500 mA g^−1^/300]	884 mA h g^−1^/1000 mA g^−1^ (ref. 105) [815.2 mA h g^−1^/1000 mA g^−1^/250]			
602.3 mA h g^−1^/200 mA g^−1^ (ref. 73) [282.2 mA h g^−1^/1000 mA g^−1^/1000]	602.3 mA h g^−1^/200 mA g^−1^ (ref. 73) [282.2 mA h g^−1^/1000 mA g^−1^/1000]	291 mA h g^−1^/100 mA g^−1^ (ref. 107) [172 mA h g^−1^/100 mA g^−1^/100]			
695 mA h g^−1^/100 mA g^−1^ (ref. 74) [245 mA h g^−1^/1500 mA g^−1^/300]	695 mA h g^−1^/100 mA g^−1^ (ref. 74) [245 mA h g^−1^/1500 mA g^−1^/300]				
352 mA h g^−1^/500 mA g^−1^ (ref. 75) [405 mA h g^−1^/500 mA g^−1^/1000]	352 mA h g^−1^/500 mA g^−1^ (ref. 75) [405 mA h g^−1^/500 mA g^−1^/1000]				
572 mA h g^−1^/100 mA g^−1^ (ref. 77) [522 mA h g^−1^/100 mA g^−1^/200]	572 mA h g^−1^/100 mA g^−1^ (ref. 77) [522 mA h g^−1^/100 mA g^−1^/200]				
668 mA h g^−1^/2000 mA g^−1^ (ref. 78) [668 mA h g^−1^/2000 mA g^−1^/1000]	1184.2 mA h g^−1^/200 mA g^−1^ (ref. 78) [668 mA h g^−1^/2000 mA g^−1^/1000]				

In Fig. 9 we plotted the capacity (mA h g^−1^) *vs.* cycle life values of CMs and metals supported flexible electrodes. By the comparing between Fig. 8 and 9, we can assume that with the increase of electrochemical cycle number the capacity of all types' electrodes is gradually decreased mainly due to the thick SEI formation, growth of Li dendrite, detachment of active materials, and structural decay of conductive supports of electrodes. In addition, from Fig. 10b the average capacity retention at the average current densities after average cycle life of CNT, CNFs, graphene, CFT, carbon coating/foam, and metals based electrodes are around 502.4, 689.65, 643.48, 705.80, 736.75, 1149.72 mA h g^−1^ at 915.38, 533.6, 1143, 1391.8, 831.25, and 655.71 mA g^−1^ corresponding to 51.65, 63.14, 77.95, 57.10, 69.80, and 71.4% of their average initial capacities after the average electrochemical cycles of 255, 393, 514, 403.33, 537, and 1010 (taking only integral number) respectively, these average values are also calculated by simple mathematical average calculation method.

As far we know limited amount of investigations have been done based on metals in the field of flexible electrodes, consequently it is hard to deciding precise approximate average values of capacity and cycle life. For instance, from ref. ^192^ without the largest value of cycle life (5000) the average cycle life drastically declined to only 345, and from ref. ^179^ without largest value of capacity (3441.9 mA h g^−1^) the average capacity value declined to 767.8 mA h g^−1^ corresponding to 62% of average initial capacity of metals supported electrodes, indicating the severe cycle instability even though this value is larger than various CMs based electrodes.

On the other hand, metals demonstrated poor adhesion strength with active materials due to plane surface and no surface functionality results in fast detachment of active materials under structural deformation. Moreover, Liang *et al.* systematically revealed that, in a specific cell 3D hierarchical CMs based electrodes enhancing the capacity retention of up to 5 times and the cycle number is 200 than that of their metallic counterparts.^205^ Furthermore, even if metals show superior electrochemical performance under flat states it will drastically fall after several times of flexion.

In brief, CMs based electrodes have more superior factors than metals-based electrodes in terms of electrochemical stability. These major advantageous factors are: (i) larger specific surface area, (ii) higher charge transfer rate for electrochemical reactions (10^8^ S m^−1^ of graphene while 10^7^ S m^−1^ of metals),^223^ (iii) better chemical stability that stimulate to wide potential window. These factors are crucial to improve the overall electrochemical performance of LIBs. Meanwhile, due to strong adhesion with active materials, CMs exhibit stable electrochemical performance under various structural deformation states. Therefore, CMs are more reliable to developing highly durable FLIBs for next-generation wearable electronic devices.

After all, from this discussion graphene-based electrodes exhibit superior electrochemical stability though various issues associated with integration of graphene in practical FLIBs applications. On the other hand, another CMs based electrodes also showed excellent electrochemical performances. Moreover, structural adaptation such as nanowire and mesh like configuration of metals will be the solution for the large mass, low surface area, and integrity of electrodes during deformation. Furthermore, controlling the pore size in metals foam-based electrodes will increase the specific surface area and electrolyte wettability. However, yet CMs and metals based flexible electrodes cannot meet the commercial demands in terms of electrochemical and mechanical performances, thus great research attentions required to implement those types of deformable electrodes in FLIBs.

## 7. Conclusion and perspectives

**Fig. 11:**
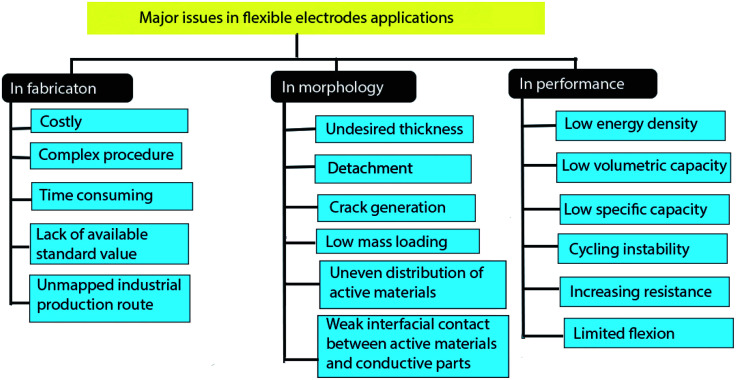
Major issues associated with fabrication, structure, and performance of flexible electrodes.

Based on recent investigations in Fig. 11, we have systematically presented the major issues associated with flexible electrodes. In addition, comparative understanding, and vital factors have been deeply discussed along with the applications of CMs and metal-based flexible electrodes for LIBs. The prominent obstacles about the electrodes are crack formation and detachment of active materials to realize desirable performance. Despite this, plenty of research has been carried out on CMs and metal-based flexible electrodes; their performances yet very poor than their rigid analogous.

Based on the above discussed experimental works, the binder-free flexible electrodes possess some excellent advantages:

(i) Firm interfacial contact between conductive parts and active materials which leads to the high charge transfer rate;

(ii) Removal of inactive binders are beneficial for reducing overall mass and unwanted heat evolution;

(iii) High mass loading due to the direct bonding between active materials and conductive materials which can enhance the overall capacity of electrodes;

(iv) Capable to retain good mechanical integrity under outer stress due to the strong binding strength between active materials and conductive parts;

(v) Direct interaction between active materials and electrolytes results in proper utilization of active materials and outstanding electrochemical performance.

Metals have better electrical conductivity than carbonaceous materials but more readily undergo corrosion and increasing sheet resistance during long-term storage. Besides, concerning CMs based electrodes, metal-based electrodes possess fast cracking and delamination of active materials. Therefore, to improve the robustness of electrodes, various techniques such as modification of the current collectors and active materials have been suggested from the materials development period. For instance, nanosized and porous morphological alteration of active materials from the spherical shaped particles ensured the great adhesion of the electrode components during deformation. Furthermore, carbonaceous materials based free-standing electrodes come into the spotlight, due to the non-existence of the current collector to avoid the detachment of the active material and current collector, permitting outstanding electrochemical performance. Another main drawback is to attaining the high rate performances while elevated power and energy densities. Possible solutions are: using electrode components with a porous and large specific surface area that enables high ionic and electronic conductivity. Utilizing carbon nanomaterials with various forms that can generate a 3D interconnected porous network, allowing a high rate of electrons and ions transportation. Moreover, hybridizing active materials NPs with a 3D carbon network increase the charge storage capacity.^224^

For the future development, it is necessary to understand some major factors such as conductivity of conductive materials, interfacial strength between active materials and conductive parts, morphological stability of conductive materials *etc.* According to our findings, we provide seven major aspects to improve the mechanical and electrochemical performance of binder-free electrodes:

(i) Selecting lightweight and ultrahigh conductive materials as charge transferring medium;

(ii) High stress tolerance limit of conductive materials under various deformation, and good chemical stability in selected electrolyte and high current density is crucial for mechanical and electrochemical stability;

(iii) As active materials bind with conductive parts for the continuing electrochemical reactions. Thus, conductive materials should be ‘deposition friendly’ for the ease of active materials deposition. Also, tight interfacial strength is a critical factor to boost the electrochemical and mechanical performance;

(iv) Well organized or super aligned deposition of micro- or nano-structured active materials is recommended for the high mass loading, proper utilizing of active materials and good interfacial contact with conductive materials. High geometrical tortuosity of current collectors can impede the homogeneous growth of active materials. So, optimal tortuosity (equal to 1) value of current collectors is beneficial for uniform active materials growth;

(v) Perfect functionalization and heteroatom doping in CMs skeletons are crucial for improving their conductivity, bond strength with active materials and mechanical flexibility;

(vi) Mesh or nanowires like formation of metals is suitable for synthesizing free-standing and binder free flexible electrodes. On the other hand, porosity controlling in mesh/nanowires or foam-based electrodes is another factor for the enhancing overall capacity;

(vii) Coating the metals current collectors with various CMs or conductive polymers is another strategy to improve the electrochemical and mechanical performance while protecting them from corrosion.

In most cases, electrodes are not able to demonstrating stable electrochemical performance under continuous mechanical deformation. Peeling-off of active materials and crack formation are common issues that limiting the commercial uses of pliable electrodes. Hybridizing active materials with a 3D carbon network or using optimized amount of appropriate binder in metals-based electrodes may help to overcome this issue. From the analysis of electrochemical behavior, metals-based electrodes possess better initial electrochemical performance but severely lowering after several number of cycles. Meanwhile, CMs based electrodes exhibit more consistency in electrochemical performance due to the greater chemical stability and higher adhesion with active materials. On the other hand, with respect to CMs, large mass, fast crack generation, and possibility of corrosion hiders the further applications of metals in deformable electrodes. Structural modification such as nanowires and 3D mesh forms of metals may help to overcome some of these issues.

More importantly the good knowledge of the electrode/electrolyte interfacial behavior under mechanical flexions contributes to better control of the electrochemical performance with mechanical deformability simultaneously. To date, many researchers have tried to predict mechanical characteristics, electrical conductivity with the energy storage capability of pliable electrodes *via* advanced computational simulation-based techniques that can help to find effective electrode materials for FLIBs.^225^ On the other side, morphology of active materials is affecting the storage capacity of electrodes such as rigid particles or nanosheets like structures of active materials cannot buffer the volume expansion during Li storing leads to delamination and crumbling of electrodes. Therefore, nanotube, or porous particle/nanosheets like formation of active materials can mitigate the self-volume expansion results in high storage capacity and long-term cycling stability.

In summary, owing to the intricate, costly, and time-consuming production route of flexible electrode materials, yet flexible LIBs could not satisfy the commercial viability. However, the lacking of industrial assessment parameters to evaluate the variations of electrochemical performances under different flexion states are impeding their desirable advancement. Though many challenges are associated with carbonaceous materials and metals based flexible electrodes for LIBs, still they containing excellent potentiality in next-generation FLIBs. Finally, based on this review, the continuous advancement in energy and power densities while maintaining favorable mechanical properties of flexible electrodes for LIBs will unquestionably increase the wearable electronic devices in real life.

## Conflicts of interest

There are no conflicts to declare.

## Supplementary Material
